# Identification of a tissue resident memory CD8 T cell-related risk score signature for colorectal cancer, the association with TME landscapes and therapeutic responses

**DOI:** 10.3389/fgene.2022.1088230

**Published:** 2023-01-04

**Authors:** Jiazheng Li, Chao Yang, Yongbin Zheng

**Affiliations:** Department of Gastrointestinal Surgery, Renmin Hospital of Wuhan University, Wuhan, China

**Keywords:** colorectal cancer, tissue-resident memory T cells, single cell RNA sequencing, prognostic model, tumor microenvironment, immunotherapy

## Abstract

**Backgrounds:** The tissue resident memory CD8 T cell (Trm) constitutes an important component of the local immunity. In the context of malignant tumors, mounting evidence also supports the potential anti-tumor property of this cell subset. Therefore, identification of Trm marker genes and exploration of the causative effect of Trm in shaping tumor microenvironment (TME) heterogeneity might provide novel insights for the comprehensive management of cancer patients.

**Methods:** By dissecting a single T cell transcriptome dataset, we acquired marker genes for Trm, which were latter applied to bulk RNA sequencing profiles of two large colorectal cancer (CRC) patient cohorts downloaded from TCGA and GEO databases. First, colorectal cancer patients were divided into different Trm clusters using consensus clustering algorithm. Then, we established a Trm-related gene (TRMRG) risk score signature and tested its efficacy in predicting prognosis for colorectal cancer patients. Moreover, a sequence of rigorous and robust analyses were also carried out to investigate the potential role of Trm-related gene risk score in tumor microenvironment remodeling and therapeutic utility of it in colorectal cancer treatment.

**Results:** A total of 49 Trm marker genes were identified by analyzing single cell RNA sequencing profiles. First, colorectal cancer patients were successfully classified into two Trm clusters with significant heterogeneity in functional enrichment patterns and tumor microenvironment landscapes. Then, we developed a Trm-related gene risk score signature and divided patients into different risk levels. High risk patients were characterized by attenuated immunogenicity, weakened sensitivity to immunotherapy, as well as adverse clinical outcomes. While low risk patients with advantages in survival exhibited increased immunogenicity, stronger metabolic activity and improved immunotherapeutic responses.

**Conclusion:** Through combinatorial analysis of single cell and bulk RNA sequencing data, the present study identified Trm to play a non-negligible role in regulating the complexity and heterogeneity of tumor microenvironment for colorectal cancer. Moreover, the Trm-related gene risk score signature developed currently was corroborated to be tightly correlated with prognosis and therapeutic responses of colorectal cancer patients, thus exhibiting potential application value for clinical practice.

## Introduction

Colorectal cancer (CRC) is the most common malignancies in digestive system with high morbidity and mortality. According to the latest statistics, CRC ranks the third and second leading causes for newly-diagnosed cancer cases and cancer-related deaths worldwide in 2020, respectively (Sung et al., 2021). Immunotherapies, with immune checkpoint inhibition (ICI) being the most representative strategy, has achieved considerably improved clinical outcomes in cancer treatment (de Miguel and Calvo, 2020). However, durable therapeutic responses can be only observed in a limited amount of cancer patient (Sharma et al., 2017). Several factors may have an impact on prognosis and immunotherapeutic sensitivity, among which tumor microenvironment (TME) landscape plays an indispensable role (Duan et al., 2020). Mounting evidence have suggested that high-level CD8 T cell infiltration was a positive sign indicating optimistic prognosis after ICI treatment (Pardoll, 2012; Bindea et al., 2013). Of all the CD8 T cell subsets, memory CD8 T cells are supposed to constitute an essential component of secondary defenses to threats to health including infections and cancers (Yenyuwadee et al., 2022). Therefore, a deep understanding of the putative role of memory CD8 T cells in shaping TME heterogeneity would be crucial to improve prognosis and optimize therapeutic strategies for cancer patients.

Traditionally, memory CD8 T cells were divided into two categories: central memory T cells (Tcm) and effector memory T cells (Tem) (Sallusto et al., 1999). As two categories of circulatory lymphocytes that move through bloodstream and lymph, Tcm and Tem are capable of migrating into peripheral tissues to provide secondary defense when reencountering antigens (Yenyuwadee et al., 2022). By contrast, tissue resident memory CD8 T cells (Trm), of which the existence was initially corroborated in the skin and intestinal mucosa of mouse models (Kim et al., 1999; Boyman et al., 2004), are non-migratory and reside permanently in within epithelial layers of peripheral tissues (Park et al., 2019). CD103 (ITGAE) and CD69 are acknowledged as two major biomarkers for Trm, which engender the cell with the ability to reside in local tissues durably and respond quickly to local antigen challenge, both are important mechanisms whereby Trm participate in local secondary defense responses (Yenyuwadee et al., 2022). In the context of malignant tumors, elegant studies have highlighted the anti-cancer property of Trm in TME, and the potential therapeutic utility of Trm in immunotherapy. For example, in lung cancer, Trm was reported to encompass higher cytotoxicity and proliferative potentials than non-Trm CD8 cells and constitute a larger proportion in patients who respond well to immunotherapies (Clarke et al., 2019). In addition, several recent works have also correlated high infiltration of Trm with prolonged survival and better sensitivity towards immunotherapy in patients with melanoma (Edwards et al., 2018), ovarian cancer (Webb et al., 2015), intrahepatic cholangiocarcinoma (Kim et al., 2021) and breast cancer (Savas et al., 2018), etc. For CRC, the specific contribution of Trm to TME landscape remains elusive.

To unravel the association between Trm and the TME characteristics for CRC patients, we first identified marker genes for Trm by dissecting single cell RNA sequencing (scRNA-seq) data. Based on the bulk RNA-seq data downloaded from the cancer genome atlas (TCGA) gene expression omnibus (GEO) database, we then used these marker genes to divide CRC patients into different clusters. In addition, more relevant genes of Trm, namely the Trm-related genes (TRMRG), were identified through differential expression analysis between Trm clusters, and were used for the construction a risk score signature. The potential application value of our TRMRG risk score signature for predicting prognosis and immunotherapeutic sensitivity was also investigated.

## Methods

### Data acquisition and processing

The analytical process of the present work was depicted in Supplementary Figure S1. In this study, GSE108989, a scRNA-seq dataset containing transcriptomes of over 10,000 single T cells from 12 CRC patients were obtained from GEO database (Zhang et al., 2018). In addition, we downloaded bulk RNA-seq and corresponding clinical profiles of two large CRC patient cohorts: TCGA-COAD/READ and GSE39582 from TCGA (https://portal.gdc.cancer.gov) and GEO (https://www.ncbi.nlm.nih.gov/geo) database, respectively. After excluding those without follow-up information, a total of 1071 CRC patients were enrolled in the present study. Baseline information of these patients were described in Supplementary Table S1. To ensure the comparability across different sequencing platforms, we converted the FPKM format of the TCGA RNA-seq data to TPM format based on previously described protocols (Conesa et al., 2016), which was believed to resemble the GEO microarray data. Moreover, two cohorts were merged into a meta-cohort after eliminating batch effects using R package “sva”.

### Acquisitoin of Trm marker genes

We focused on single T cell RNA-seq data in GSE108989 to identify Trm marker genes. R package “Seurat” was employed for dimension reduction. Based on the false discovery rate (FDR) <0.05 criterion, dimensions with significant separation were identified through principal component analysis (PCA). The top 20 principle components (PCs) were utilized to divide cells into different clusters *via* the t-distributed stochastic neighbor embedding (tSNE) algorithm. First, CD4 and CD8 T cells were separated based on their own markers, with CD4 representing CD4 T cells, CD8 and CD8A for CD8 T cells. Then, we screened out Trm cell subsets among the CD8 cell populations based on known marker genes for Trm and genes used in Zhang’s work (Zhang et al., 2018). Finally, we selected the marker genes for each T cell subset using the |log2 [FC (fold change)]| > 1 and FDR <0.05 criterion.

### Functional enrichment analysis and consensus clustering analysis

To gain an insight into the pathways and biological function enriched in the Trm marker genes selected above, we sought to Kyoto Encyclopedia of Genes and Genomes (KEGG) and Gene Ontology (GO) functional enrichment analysis using “clusterprofiler” package in R. Then, based on the expression pattern of these marker genes, we attempted to divide CRC patients in meta-cohort into different clusters using “ConsensusClusterPlus” package in R. The optimal clustering number was determined by k-means algorithm and cumulative distribution function (CDF) curve. The stability of our clustering was evaluated by PCA analysis.

### Clinical correlation analysis of Trm clusters

To investigate the association between different Trm clusters and clinical features, we analyzed differences in the distribution of clinicopathological factors including age, gender, T,N,M status and tumor stage within distinct CRC Trm clusters using chi-square test or Fisher’s precision probability test.

## Correlation of CRC Trm clusters with biological function and TME

For identification of biological functions enriched in different Trm clusters, we downloaded KEGG pathway gene set and Hallmark biological signature gene set from MsigDB (http://www.gsea-msigdb.org) database. Gene set variation analysis (GSVA) based on the two gene sets were performed to calculate the enrichment score (ES) of each CRC sample in TCGA cohort. Variations in ES between different Trm clusters were displayed in the form of heatmap and bar plot, using R packages “pheatmap” and “ggplot2”, respectively. To investigate the TME characteristics within different Trm clusters, we sought to the Estimation of STromal and Immune cells in MAlignant Tumor tissues using Expression data (ESTIMATE) algorithm (Yoshihara et al., 2013) to calculate the proportion of immune cell and stroma content in TME, along with the tumor purity, and compared their differences between different Trm clusters. Moreover, marker genes of 28 immune cell types were obtained from Charoentong’s paper (Charoentong et al., 2017) and further subjected to single sample gene set enrichment analysis (ssGSEA) for estimation of the infiltration levels of 28 immune cells.

### Identification of gene subtypes based on TRMRG

To find out more TRMRG, we screened out genes differentially expressed between various Trm clusters using “limma” package in R. The inclusion criteria were log |FC |>0.585 and adjusted *p*-value < 0.05. Then, based on the expression pattern of TRMRG, we performed unsupervised consensus clustering and classified CRC patients into different TRMRG subtypes.

### Construction and validation of TRMRG risk score signature

To quantify CRC patients’ risk level, we developed a TRMRG risk score signature. First, all CRC patients in meta-cohort were randomized into a training (*n* = 536) and validation (*n* = 535) cohort at 1:1 ratio, and training cohort was used to conduct univariate Cox regression analysis to primarily filter out prognosis-related genes. Then, to reduce the overfitting risk, we employed least absolute and selection operator (LASSO) regression model on genes selected above. Eligible genes were finally subjected to multivariate Cox regression analysis for risk score calculation. The formula for risk score estimation was:

$$Risk\;score=\sum \left(\mathrm{Exp}\;i\;*\;Coef\;i\right)$$

Exp i and Coef i represented the expression and multivariate Cox coefficient of each gene. The stratification of TCGA patients’ risk level was based on the median value of TRMRG risk score. Subsequently, the risk score signature was applied to validation cohort, TCGA cohort, GEO cohort as well as the whole meta-cohort, respectively, for validation. Kaplan-Meier plots and time-dependent receiver operating curves (ROC) were drawn to estimate the predictive efficacy of TRMRG risk score signature.

### Clinical correlation analysis of TRMRG risk score signature

To investigate the clinical significance of TRMRG risk score signature, we first compared the differences in distributions of different clinicopathological factors between high and low risk CRC patients. Next, we stratified patients into different clinical subgroups according to age (≤65 vs. >65), gender (male vs. female), tumor stage (I-II vs. III-IV), tumor location (left vs. right), T status (T1-T2 vs. T3-T4), N status (N0 vs. N1-N3), M status (M0 vs. M1), and compared the risk score differences quantitatively.

### Correlation of TRMRG risk score signature with biological function and TME

We employed gene set enrichment analysis (GSEA) to explore the correlation of risk score with biological function. To figure out whether patients at different risk level harbor markedly varied TME landscapes, comparisons of ESTIMATE score and immune cell infiltration were performed. Notably, anti-cancer immune response is a multi-step cyclic event which can be conceptualized into seven main steps including release of cancer cell antigens (step 1), cancer antigen presentation (step 2), priming and activation (step 3), trafficking of immune cells to tumors (step 4), infiltration of immune cells into tumors (step 5), recognition of cancer cells by T cells (step 6), and killing of cancer cells (step 7) (Chen and Mellman, 2013). Representative genes involved in each step were curated from tracking tumor immunophenotype (TIP) website and formed seven gene sets (Xu et al., 2018). We used ssGSEA algorithm to estimate the activation degree of the seven steps and made comparisons between high and low risk patients (Du et al., 2022).

### Expression of HLA family genes and inhibitory molecules, TCR richness analysis of TRMRG risk score signature

We compared the expression differences of HLA family genes (Wang et al., 2022) and immunosuppressive molecules (Pitt et al., 2016; Song et al., 2018) between high and low risk CRC patients. Moreover, profiles for the richness and diversity of T cell receptor (TCR) repertoire were obtained from TCGA database and used for difference comparison between high and low risk patients (Song et al., 2022).

### Immunotherapeutic sensitivity analysis of TRMRG risk score signature

Given that a large number of established immune-related risk models showed potential utility in guiding immunotherapeutic options (Li X. et al, 2022; Gao et al., 2022; Xu et al., 2022), we determined to continue our research in this direction. First, the expression levels of 3 commonly-used target genes for immune checkpoint inhibitor (ICI) therapies, CTLA-4, PD-L1 and PD1 were compared between high and low risk patients. Next, Tumor Immune Dysfunction and Exclusion (TIDE) scoring, a parameter inversely correlated with ICI efficacy was estimated in each CRC patient according to the instruction on TIDE website (http://tide.dfci.harvard.edu/) (Jiang et al., 2018). In addition, the immunophenoscore (IPS) for predicting anti-PD1 and anti-CTLA4 therapeutics among TCGA CRC patients was obtained from the Cancer Immunome Atlas (TCIA, https://tcia.at/home). Generally speaking, higher IPS represents better accuracy for the more corresponding result (Jiang et al., 2021; Guo et al., 2022). We divided patients into high (9, 10), medium (7, 8) and low (5, 6) IPS groups and analyzed the frequencies of three IPS groups in high and low risk patients, respectively. Finally, to validate our conclusions drawn from the analysis of ICI-related parameters, we also directly applied the TRMRG risk score signature to two external immunotherapeutic cohorts: IMvigor210 for urothelial carcinoma patients receiving anti-PD-L1 treatment, and GSE78220 for melanoma patients with anti-PD1 administration.

### Correlation of TRMRG risk score signature with metabolism

In a 2020 study, Yang et al. (2020) integrated over 2000 currently-identified metabolism-related genes and developed a gene set signature containing 115 items. Herein, we selected gene sets correlated with carbohydrate, lipid and amino acid and drug metabolism and estimated the ES of them for CRC patients using “ssGSEA” algorithm. Comparisons of the ES were made between high and low risk groups. Besides, we employed consensus clustering analysis in an attempt to classify CRC patients into different metabolic subtypes. Risk score variations within different metabolic subtypes were also compared to further elucidate TRMRG risk score’s association with metabolism.

### Chemotherapeutic sensitivity analysis

To select the appropriate anti-tumor medicine for CRC patients at different risk levels, we used R package “pRRophetic” to estimate the half inhibitory concentration (IC50) values for several chemotherapeutic drugs and compared IC50 differences between high and low risk patients latter. In addition, we downloaded drug sensitivity data to more than 20,000 compounds of the NCI-60 cell line from the Cell Miner database (Reinhold et al., 2012). Missing values of drug sensitivity data was supplemented by “impute.knn” function of the “impute” package in R. Then, drugs with the clinical use being approved by FDA was selected to perform Pearson correlation analysis to find out the correlation of gene expression with IC50 value.

### External validation of TRMRG risk score signature in predicting prognosis and therapeutic responses

To testify the stability of TRMRG risk score signature in predicting prognosis and therapeutic responses, two external CRC cohorts: GSE17536 and GSE17537 were downloaded from GEO database for external validation. Risk score calculation and risk level stratification followed the same criteria as the meta-cohort. We conducted Kaplan-Meier analysis and time-dependent ROC analysis to test the efficacy of TRMRG risk score for predicting OS in this two cohorts. Moreover, TIDE algorithm was also applied to validate the association of risk score with sensitivity to immunotherapies in these two cohorts.

### Development of a nomogram based on TRMRG risk score signature

To strengthen the clinical utility of TRMRG risk score signature, a nomogram integrating risk score and other clinicopathological factors to predict patient’s survival was developed using R package “rms”. Meanwhile, we depicted calibration curves and conducted time-dependent ROC and decision curve analysis (DCA) to evaluate the predictive efficacy of the nomogram.

### Statistical analysis

All statistical analyses were performed in R version 4.1.2. Comparisons of numerical variables used Wilcoxon rank sum test. For survival data, log-rank test was chosen and the result was display in the form of Kaplan-Meier plots. The predictive efficacy of variables was demonstrated by time-dependent ROC curves with area under curves (AUC) values.

## Results

### Identification and functional analysis of trm marker genes

**FIGURE 1:**
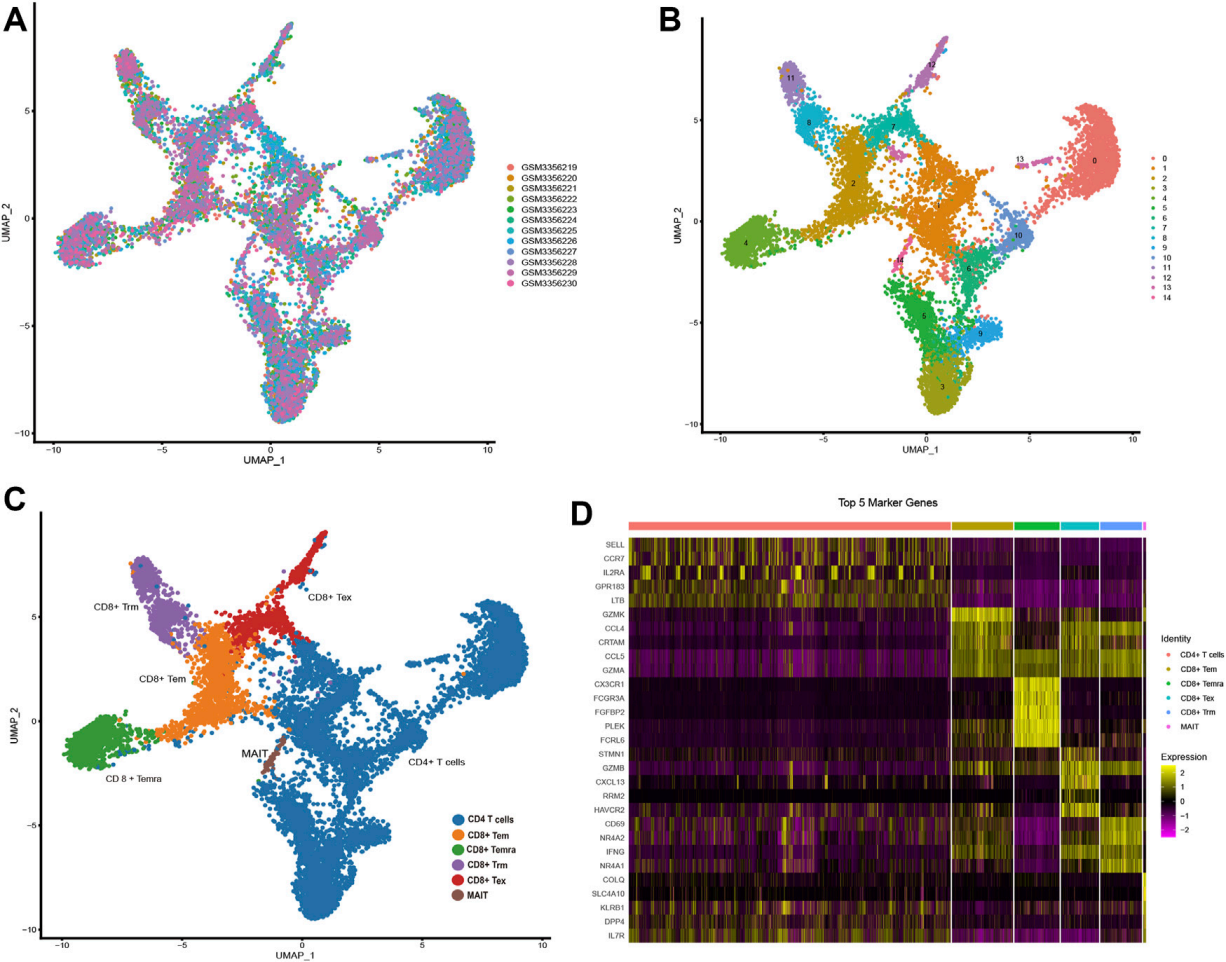
Identification of Trm marker genes *via* scRNA-seq analysis **(A,B)** t-SNE plots colored by different **(A)** CRC samples and **(B)** cell clusters. **(C)** Identification of different T cell subsets. **(D)** Heatmap showing the top 5 marker genes of each T cell subtype. Tex: exhausted T cell; Tem: effector memory T cell; Temra: recently-activated effector memory T cell; MAIT: mucosal-associated invariant T cell; Trm: tissue-resident memory T cell.

GSE108989 comprises transcriptomes of 11,138 single T cells from 12 CRC patients (Figure 1A). After performing dimension reduction using the top 20 PCs, 15 cell clusters were obtained (Figure 1B), with cluster 0, 1, 3, 5, 6, 9, 10, 13 representing CD 4 T cells, and cluster 2, 4, 7, 8, 11, 12, 14 representing CD 8 T cells. Among CD 8 cell populations, cluster 8 and 11 were recognized as Trm subsets by referring to known Trm marker genes and genes used by Zhang’s work (Zhang et al., 2018) (Figure 1C, Supplementary Figure S2). Marker genes for each cell subset were listed in Supplementary Table S2. The heat map in Figure 1D depicted the top 5 marker genes for each T cell subset. Of note, a total of 49 marker genes for Trm were identified. Functionally, these genes were tightly associated with the regulation of immune cell activity, MHC protein complex and antigen processing (Supplementary Figure S3A).

### Genetic alteration landscape of Trm marker genes in TCGA cohort

Among all the 49 Trm marker genes, the expression data of 42 genes could be obtained in both TCGA and GEO cohort. It was demonstrated that 31 of these genes were differentially expressed between tumor and normal tissues (Supplementary Figure S4A). As shown in Supplementary Figure S4B, significant variations in somatic copy number of these marker genes could also be observed. Of note, the variation tendency of gene expression and copy number variation (CNV) alteration was the same for some genes (e.g., CD69, GPR65, FOSB with decreased expression in tumors also exhibited CNV loss; CCL4, CAPG highly expressed in tumors also exhibited CNV gain), while was the opposite for others (e.g., CCL5, PTGER4, CD160 with decreased expression in tumors were associated with CNV gain; JUN with increased expression in tumors were associated with CNV loss) The chromosome location of CNVs was shown in Supplementary Figure S4C. Based on the waterfall plot in Supplementary Figure S4D, 24.4% samples in TCGA cohort had mutations in Trm marker genes. Among the mutated genes, EGR4, IL18RAP and NR4A2 shared the highest mutation frequencies. The expression correlation and prognostic value of Trm marker genes were depicted in a comprehensive network (Supplementary Figure S4E). It was suggested that the expression of most genes were positively correlated with each other. Besides, eight of these genes including GZMB, IVNS1ABP, EGR2, MAP3K8, STOM, ZFP36, PFKFB3 and HOPX were identified to be prognosis-related genes according to univariate Cox analysis, with GZMB associated with prolonged OS and the rest indicating impaired prognosis. Based on the above analysis, the expression of Trm marker genes were characterized by significant variations between tumor and normal tissues, along with frequent CNV changes and mutation events, which highlighted the potential regulatory functions of these genes in CRC tumorigenesis.

### Identification of Trm clusters for CRC patients

**FIGURE 2:**
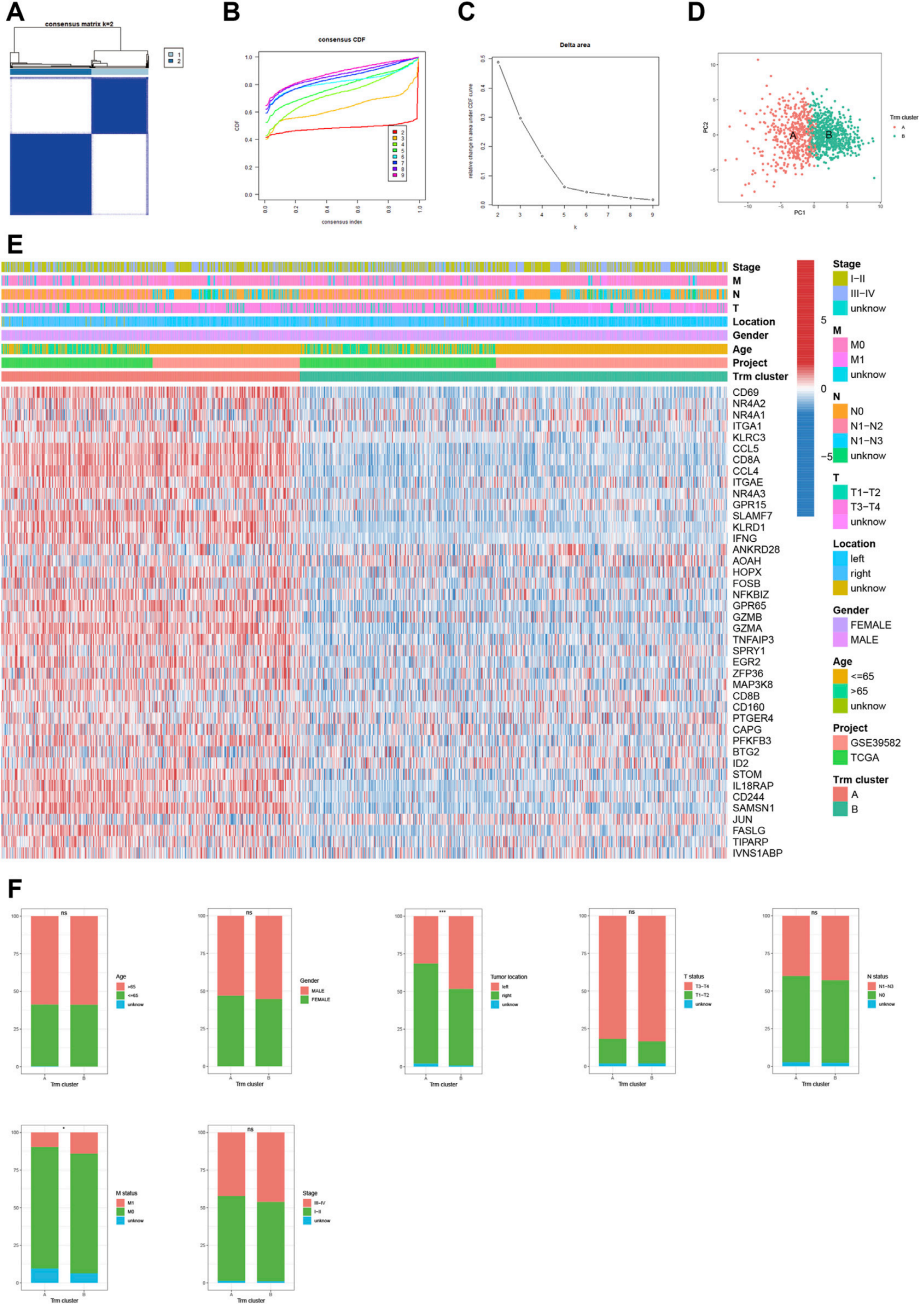
Identification of Trm clusters in CRC patients. **(A)** Consensus matrix heatmap showing two Trm clusters. **(B)** CDF curve, **(C)** k-mean algorithm and **(D)** PCA analysis showing the robustness and stability of the clustering. **(E)** Heatmap showing the expression pattern of Trm marker genes in two clusters. **(F)** Bar plots showing the correlation of Trm clusters with clinicopathological factors including age, gender, tumor location, T, N, M status and tumor stage. CDF: Cumulative distribution function; PC: Principal component. Statistical Significance: *p < 0.05; ***p < 0.001; ns: not significant.

To shed light on the expression pattern of Trm marker genes in large CRC patient population, we conducted unsupervised consensus clustering analysis for patients of meta-cohort (Figure 2A). Based on CDF curve (Figure 2B) and k-means algorithm (Figure 2C), the optimal clustering number was 2. PCA analysis further validated the stability of the clustering (Figure 2D). According to the expression pattern of Trm marker genes, we defined cluster A as high-Trm and cluster B as low-Trm subtypes, respectively (Figure 2E). In terms of clinical relevance, the proportion of CRC patients with left-sided and metastatic tumors (M1 status) was markedly larger in Trm cluster B than that in Trm cluster A (Figure 2F). While there were no significant differences in the distributions of age, gender, T, N status, and tumor stage between two Trm clusters.

### Correlation of Trm clusters with biological function and TME

**FIGURE 3:**
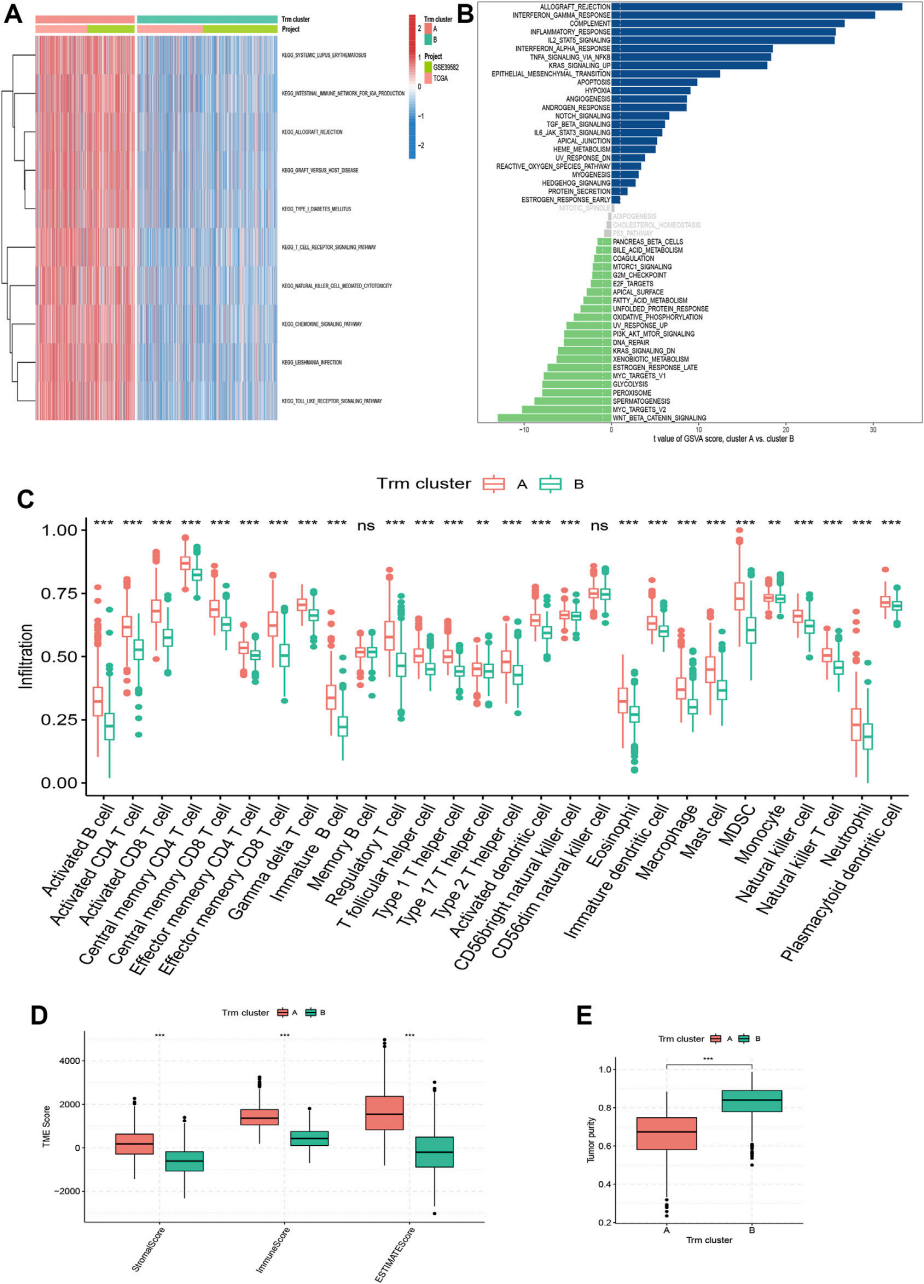
Correlation of Trm clusters with biological function and TME landscapes. **(A)** Heatmap showing the enrichment pattern of KEGG items for two Trm clusters. **(B)** Bar plot showing the enrichment pattern of Hallmark items for two Trm clusters. **(C)** Box plots comparing the infiltration differences of 28 immune cells between two Trm clusters. **(D,E)** Box plots comparing the differences in **(D)** TME score and **(E)** tumor purity between two Trm clusters. TME: Tumor microenvironment; KEGG: Kyoto Encyclopedia of Genes and Genomes. Statistical Significance: **p < 0.01; ***p < 0.001; ns, not significant.

To gain further insight of biological functions enriched in different Trm clusters, we performed GSVA analyses based on KEGG and Hallmark gene sets, respectively. For KEGG gene set, cluster A had a higher enrichment level of immune-related pathways (Figure 3A). For Hallmark gene set, biological processes in relation to immune-activation (e.g., allograft rejection, interferon alpha and interferon gamma response, inflammatory response, etc.) and stromal e.g., hypoxia, angiogenesis and epithelial mesenchymal transition (EMT) activities were all significantly enriched in Trm cluster A (Figure 3B). In addition, an overwhelming majority of immune cells were more densely populated in the TME of Trm cluster A (Figure 3C). Besides, patients of Trm cluster A were associated with enhanced immune, stroma and ESTIMATE scores (Figure 3D), along with a lower tumor purity, as compared to patients with Trm cluster B (Figure 3E). Therefore, Trm cluster A was associated with abundant immune cells and stromal contents, along with declined tumor purity. By contrast, Trm cluster B was characterized by enhanced tumor purity and a paucity of immune and stromal contents in TME. Notably, Wnt_β-catenin pathway, a canonical carcinogenic pathway for malignancies in digestive system (Xue et al., 2021; Wen et al., 2022; Yang et al., 2022; Zhang et al., 2022; Zhu et al., 2022), was the Hallmark item with the highest enrichment level in Trm cluster B (Figure 3B), which reflected the high tumor purity of this cluster.

### Identification of gene subtypes based on TRMRGs

Based on the log |FC|>0.585, adjusted *p*-value < 0.05 standard, a total of 806 TRMRGs were identified to be differentially expressed between two Trm clusters, of which 742 and 64 genes were up-regulated in cluster A and B, respectively (Supplementary Table S3). According to KEGG and GO analysis, these TRMRGs were functionally enriched in immune-related pathways and biological signatures, similar to the enrichment pattern of Trm marker genes (Supplementary Figure S3B, C). Subsequently, we identified two gene subtypes based on the expression of TRMRG (Supplementary Figure S5A–D). Similar to Trm clusters, the two TRMRG subtypes also encompassed totally distinct TME landscapes. With up-regulated TRMRG expression, subtype A could be regarded as an alternative to Trm cluster A, which was characterized by abundant immune cell and stromal contents, as well as lower tumor purity in TME. In contrast to it, TRMRG subtype B demonstrated a paucity in immune and stromal contents in TME but elevated tumor purity, and represented Trm cluster B (Supplementary Figure S5E–G).

### Construction of TRMRG risk score signature

To quantify CRC patients’ risk level using TRMRG, we conducted univariate Cox regression analysis for patients in training cohort and primarily selected 228 genes with prognostic indication value (Supplementary Table S4). Of note, 12 genes remained when further subjected to LASSO Cox regression analysis (Supplementary Figure S6A, B). Finally, seven eligible genes were screened out through multivariate Cox regression analysis, including five high risk (CYTH4, ADAP2, DAPK1 and SPARCL1) and three low risk (CXCL13, CCL22 and RTP4) genes (Supplementary Figure S6C). Notably, expressions of seven TRMRGs were positively correlated with two well-defined Trm marker genes: ITGAE and CD69, suggesting the intimate association with Trm (Supplementary Figure S7). Risk score was calculated based on the result of multivariate Cox regression analysis.

Risk score = (−0.227 * expression of CXCL13) + (0.396 * expression of CYTH4) + (0.442 * expression of ADAP2) + (0.312 * expression of DAPK1) + (0.138 * expression of SPARCL1) + (−0.509 * expression of CCL22) + (−0.246 * expression of RTP4).

Based on the median risk score, patients in training cohort were divided into high risk (*n* = 268) and low risk (*n* = 268) groups. Risk score’s distribution, survival status for high and low risk patients were shown in Supplementary Figure S6D. Moreover, the expression pattern of seven TRMRGs between high and low risk groups was depicted in the heatmap in Supplementary Figure S6E. Kaplan-Meier plots correlated high risk patients with impaired OS (log rank *p* < 0.01, Supplementary Figure S6F). Besides, the predictive efficacy of TRMRG risk score signature was also corroborated by time-dependent ROC analysis, as AUC for 5, 7 and 10 years OS were 0.755, 0.756 and 0.740, respectively (Supplementary Figure S6G).

### Validation of TRMRG risk score signature

To verity the generalizability of TRMRG risk score signature for CRC patients, we further applied it to the test cohort, TCGA cohort, GSE39582 and the meta-cohort, respectively (Supplementary Figures S8–S11). The formula for risk score calculation and the cut-off value for risk level stratification were consistent with that of the training cohort. With satisfaction, advantages in OS still exists for low risk patients. More importantly, TRMRG risk score maintained its high accuracy in prognosis prediction according to time-dependent ROC analysis, as the AUCs for predicting 5, 7 and 10 years OS were all over 0.6 in these cohorts.

### Clinical correlation analysis of TRMRG risk score signature

**FIGURE 4:**
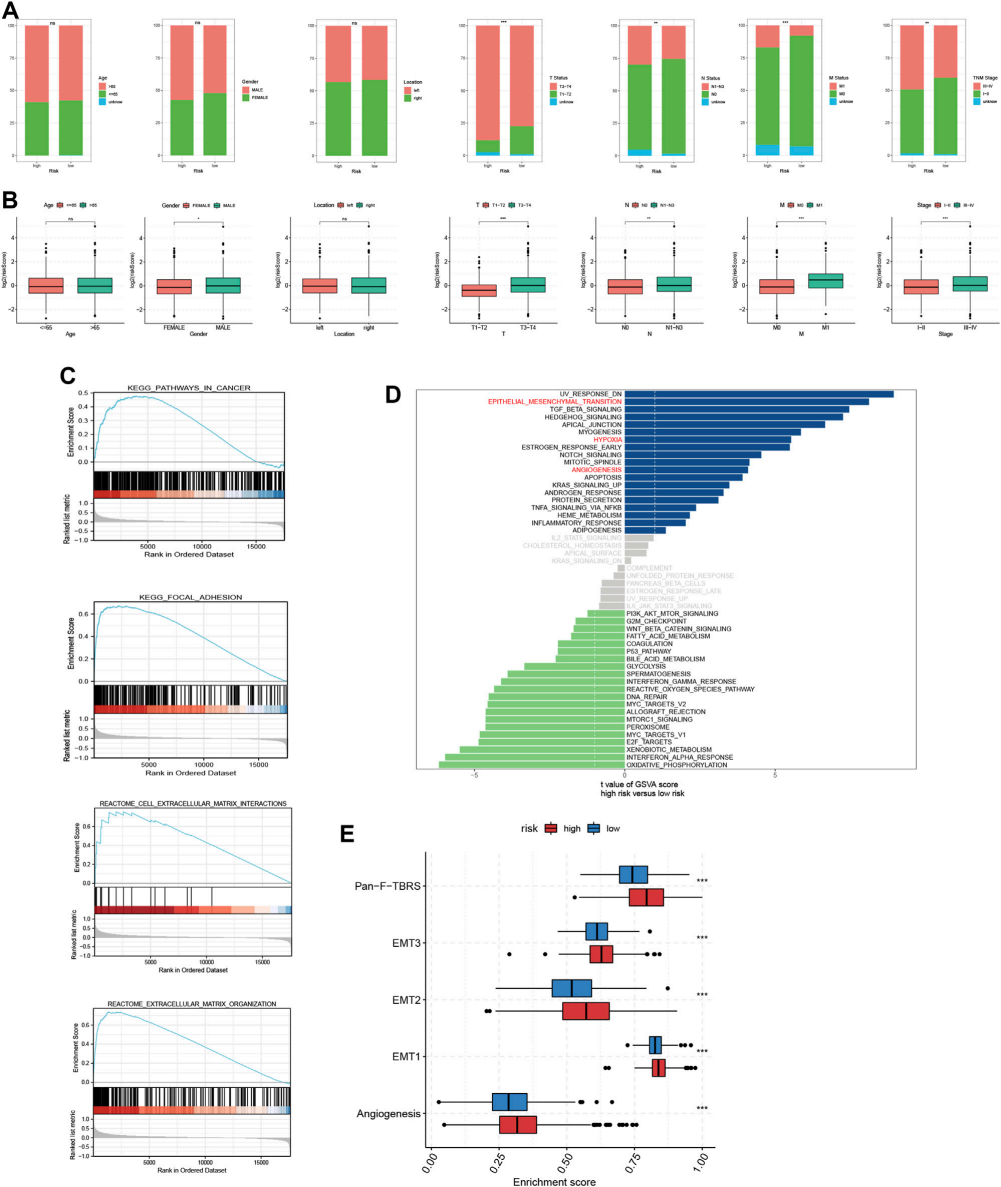
Analysis of clinical correlation and biological function for TRMRG risk score signature. **(A)** Bar plots showing the frequencies of different clinical features including age (>65 vs. ≤65), gender (male vs. female), tumor location (left vs. right), T status (T1-T2 vs. T3-T4), N status (N0 vs. N1-N3), M status (M0 vs. M1) and tumor stage (I-II vs. III-IV) in high and low risk patients. **(B)** Box plots comparing risk score differences between multiple clinical subgroups. **(C)** GSEA analysis of TRMRG risk score signature. **(D)** Bar plot showing the enrichment pattern of Hallmark items for high and low risk patients. **(E)** Box plots comparing enrichment differences of gene sets developed by Mariathasan et al. Statistical Significance: *p < 0.05; **p < 0.01; ***p < 0.001; ns, not significant.

We classified CRC patients from meta-cohort into several clinical subgroups to investigate the clinical correlation of TRMRG risk score signature. As shown in Figure 4A, there were higher frequencies of patients with advanced clinical features including T3-T4 status, M1 status, N1–N3 status and stage III-IV being classified into high risk group, while no association was found between risk level and age, gender and tumor location. Then we removed patients with unknown clinical information and compared the risk score differences between different subgroups. It was revealed that patients with advanced T, N and M status as well tumor stages all exhibited heightened risk scores (Figure 4B). Therefore, our present analysis correlated high risk patients with more advanced clinical features.

### Correlation of TRMRG risk score signature with biological function and TME

**FIGURE 5:**
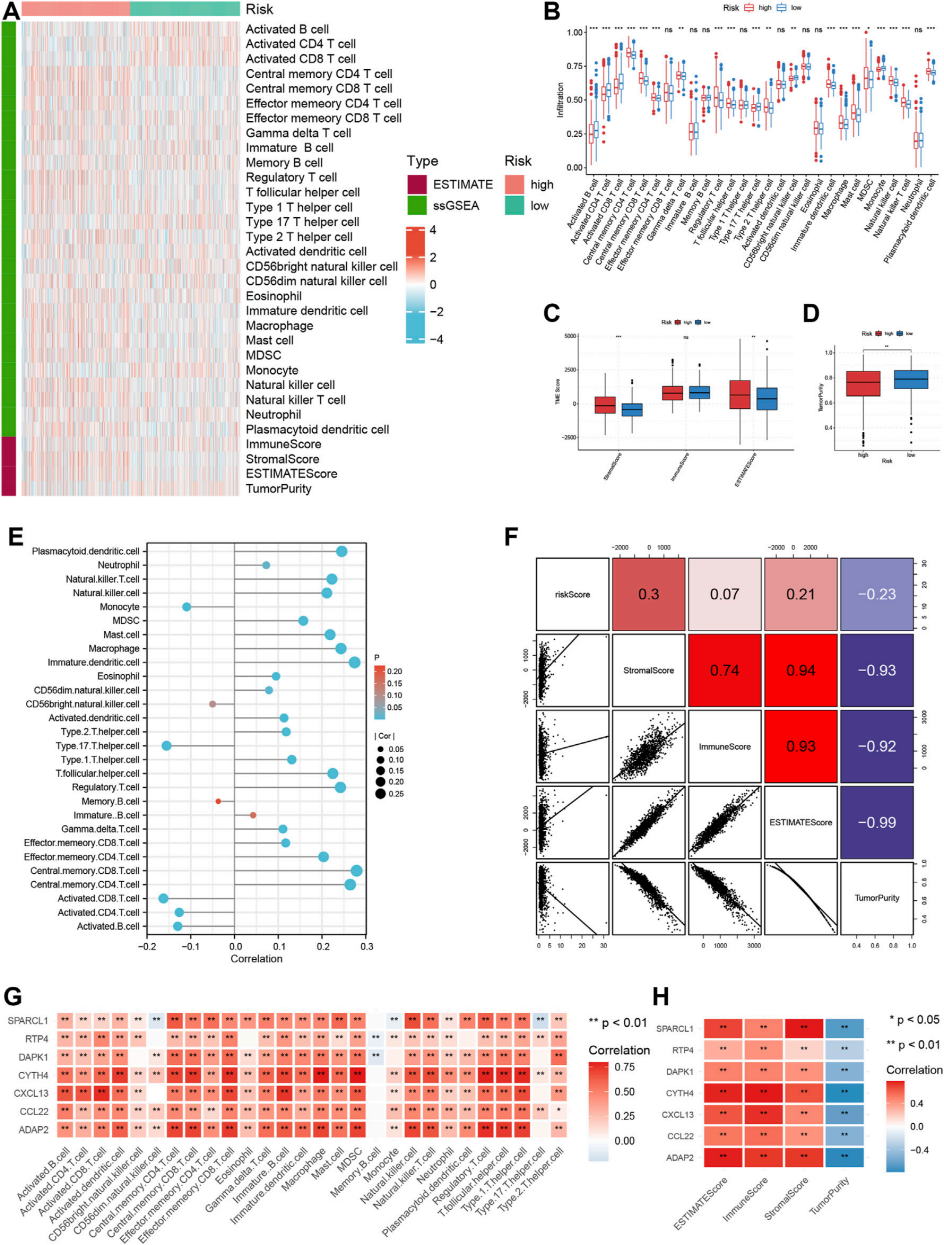
Analysis of TME landscape for TRMRG risk score signature. **(A)** Heatmap showing the immune cell infiltration level, TME score and tumor purity between high and low risk patients. **(B–D)** Box plot comparing differences in **(B)** immune cell infiltration level **(C)** TME scores and **(D)** tumor purity between high and low risk patients. **(E,F)** Pearson correlation analysis of the risk score with **(E)** immune cell infiltration levels, **(F)** TME scores and tumor purity. **(G,H)** Pearson correlation analysis of expressions of individual TRMRG with **(G)** immune cell infiltration levels, **(H)** TME scores and tumor purity. Statistical Significance: *p < 0.05; **p < 0.01; ***p < 0.001; ns: not significant.

According to GSEA functional enrichment analysis, risk scores were positively associated with carcinogenic activities and extracellular matrix (Figure 4C). Besides, Hallmark items such as hypoxia, epithelial mesenchymal transition, angiogenesis (Figure 4D), and gene signatures (EMT1, EMT2, EMT3, angiogenesis and pan-F-TBRS) developed by Mariathasan et al. (2018) were all highly enriched in high risk patients (Figure 4E), suggesting that high risk patients were characterized by enhanced stromal activities. Next, ESTIMATE and immune infiltration analyses further revealed the heterogeneity in TME landscapes between high and low risk patients. As shown in Figures 5A, B, activated CD4 T cells, CD8 T cells and B cells had higher enrichment levels in low risk patients than in high risk patients, while memory lymphocytes and multiple types of immunosuppressive cells including myeloid-derived suppressor cells (MDSC), regulatory T cells (Treg), immature dendritic cells (DC) and Th2 cells were more abundant in high risk patients (Figures 5A, B). Then, based on ESTIMATE analysis, high risk patients were associated with increased stromal scores (Figures 5A, C) and decreased tumor purity (Figures 5A, D). In addition, the risk score showed positive correlations with a vast majority of immune cells (Figure 5E) and TME scores (Figure 5F) while inversely associated with tumor purity (Figure 5F). For each individual TRMRG, it was found that their expressions were positively correlated with infiltrations of most immune cells and TME scores, while inversely correlated with tumor purity (Figures 5G, H).

**FIGURE 6:**
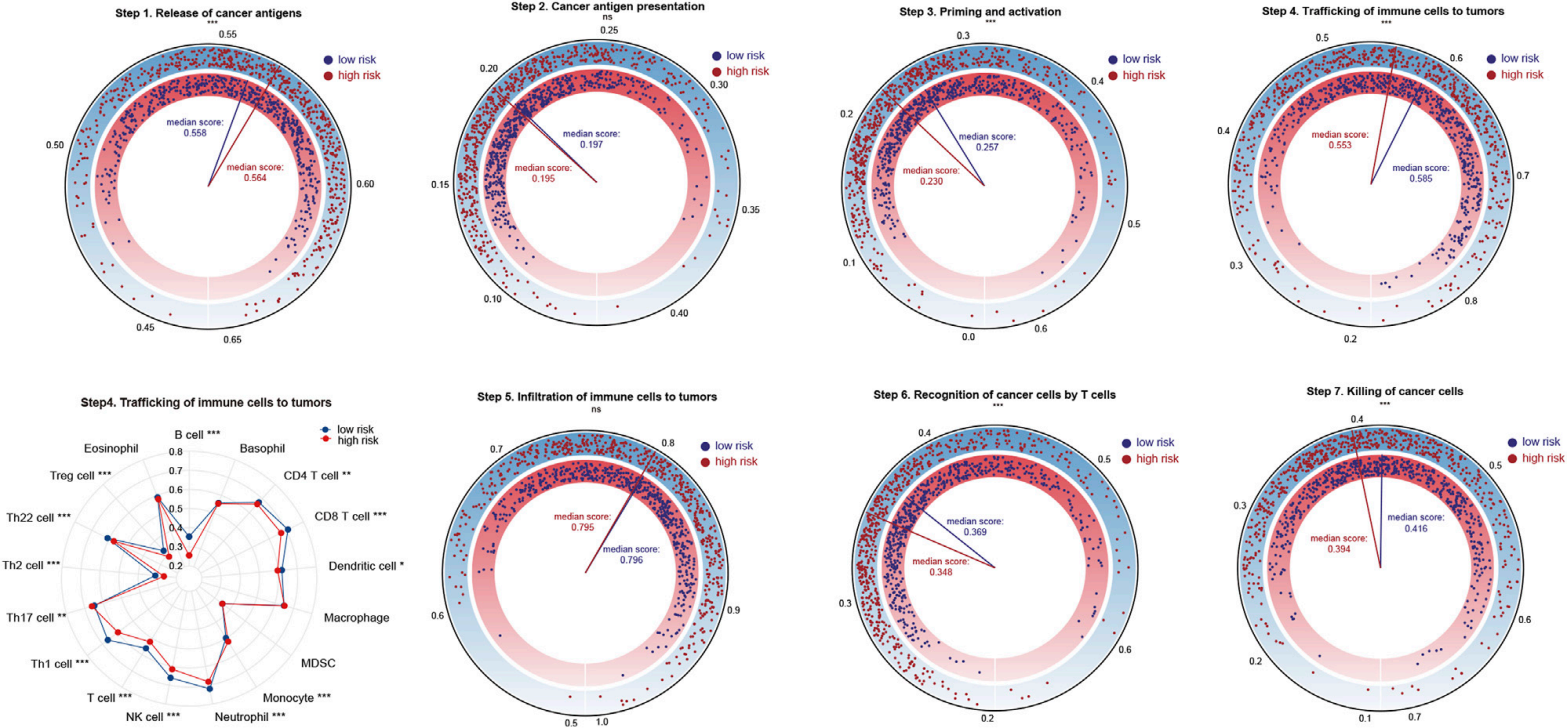
Analysis of the activation status of seven-step anti-cancer immunity cycle for high and low risk patients. Statistical Significance: *p < 0.05; **p < 0.01; ***p < 0.001; ns, not significant.

Next, we sought to compare the activation degree of each step of the anti-cancer immune response between high and low risk patients. As shown in Figure 6, the ability of immune priming and activation (step 3), T cells’ recognition (step 6) and killing (step 7) of cancer cells were all stronger in low risk patients. However, high risk patients were more competent in the release of cancer antigen (step 1). Generally speaking, low risk patients were more capable of trafficking immune cells to tumors (step 4). In detail, the trafficking ability of the vast majority of immune cells such as B cells, CD4 and CD8 T cells, neutrophils, etc., was stronger in low risk patients, whereas high risk patients showed increased trafficking ability of Th17 cells and monocytes.

### Expression of HLA family genes and immunosuppressive molecules, TCR richness analysis of TRMRG risk score signature

**FIGURE 7:**
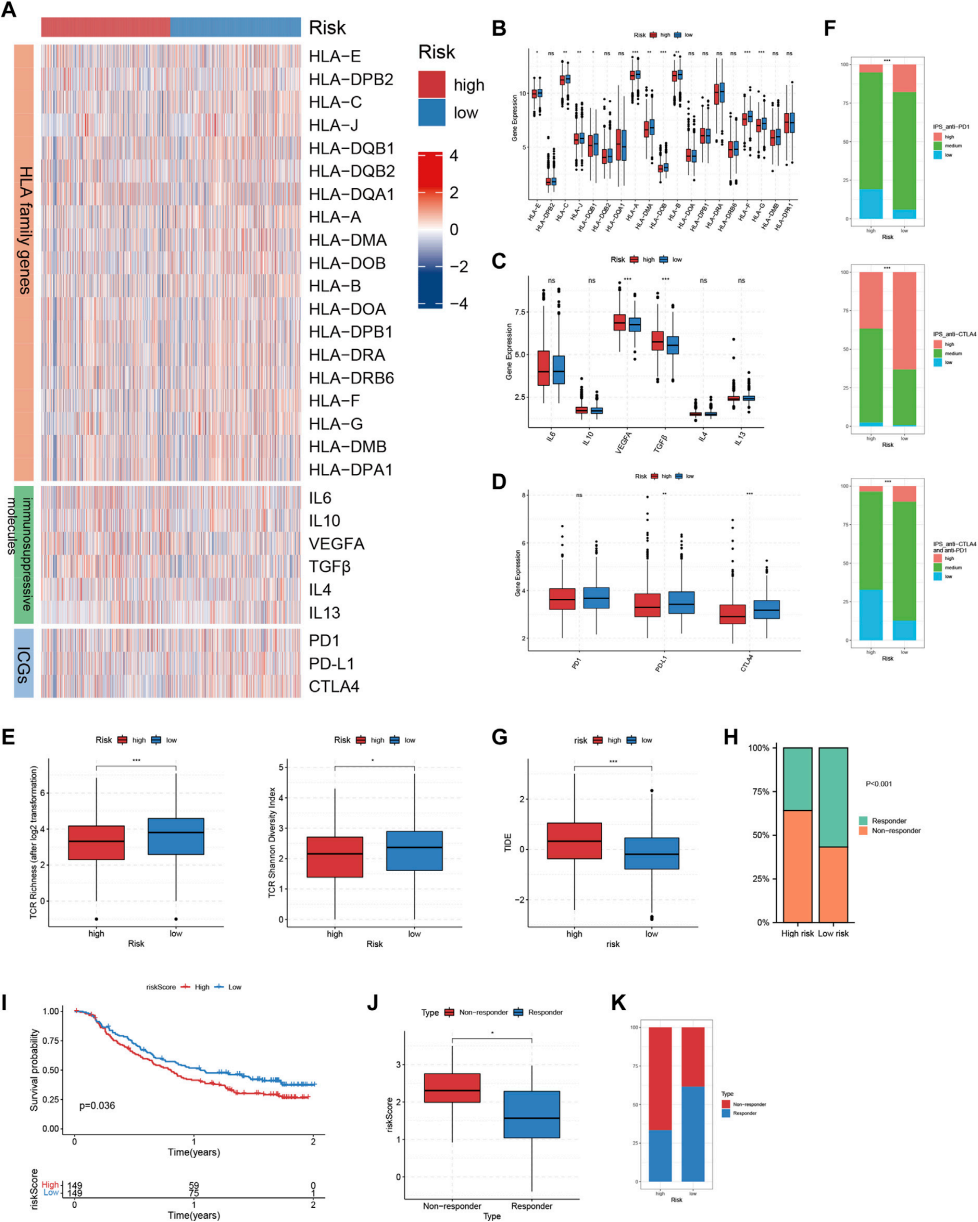
Immunotherapeutic sensitivity analysis of TRMRG risk score signature. **(A)** Heatmap showing the expression patterns of HLA family genes, immunosuppressive molecules and ICGs between high and low risk patients. **(B–D)** Bar plots comparing expression differences of HLA family genes, immunosuppressive molecules and ICGs between high and low risk patients. **(E)** Differences in TCR richness and diversity between high and low risk patients. **(F)** Bar plots showing the frequencies of high, medium, low level IPS scores for anti-PD1 treatment, anti-CTLA4 treatment and the combinatorial therapy in high and low risk patients. **(G)** Box plot comparing differences in TIDE scores between high and low risk patients. **(H)** Frequencies of responders and non-responders to ICI therapies for patients with high and low risk. **(I)** Kaplan-Meier plot showing the OS for high and low risk patients after receiving anti-PD-L1 treatment in IMvigor210 cohort. **(J)** Box plot comparing the risk scores for responders and non-responsers in GSE78220. **(K)** Frequencies of responders and non-responders in high and low risk group in GSE78220. Statistical Significance: *p < 0.05; **p < 0.01; ***p < 0.001; ns: not significant.

We compared the expressions of HLA family genes and inhibitory molecules, as well as TCR repertoire richness between high and low risk patients. Of the 19 HLA family genes, 10 were found to be highly-expressed in low risk patients (Figures 7A, B). The expression of two immunosuppressive molecules, VEGFA and TGFβ was significantly up-regulated in high risk patients compared with that in low risk patients (Figures 7A, C). In addition, low risk patients harbored increased TCR richness and diversity (Figure 7E). Taken together, our analysis correlated an immune-activated TME with low risk patients, while on the contrary, an immunosuppressive TME with high risk patients.

### Immunotherapeutic sensitivity analysis of TRMRG risk score signature

The above analysis revealed prominent heterogeneity in TME landscapes between high and low risk patients. As the efficacy of ICI therapy is largely dependent on the interaction of various components within TME, we determined to conduct multi-omic analysis exploring the association between risk score and immunotherapeutic responses. First, up-regulation of PD-L1 and CTLA4, which were two immune checkpoint genes as well as two commonly-used targets for ICI treatment, could be found in low-risk patients (Figures 7A, D). Then, high-level IPS scores for positive responses to both anti-CTLA4 and anti-PD1 treatment, as well as the combinatorial therapy, all accounted for larger proportions in patients with low risk (Figure 7F). In addition, high risk patients were characterized by significantly higher TIDE scores (Figure 7G). Likewise, the non-responders to ICI treatment predicted by TIDE algorithm took up a larger proportion in high risk group (Figure 7H). Finally, we applied the TRMRG risk score signature to 2 ICI treatment cohorts for external validation. For IMvigor210, extended OS after receiving anti-PD1 treatment could be observed in low risk patients (Figure 7I). For GSE78820, responders to ICI treatment exhibited lower risk scores (Figure 7J) and constituted a larger portion in low risk group (Figure 7K). Based on the analysis above, it was concluded that low risk patients were supposed to gain benefit from ICI treatment.

### Correlation of TRMRG risk score signature with metabolism

**FIGURE 8:**
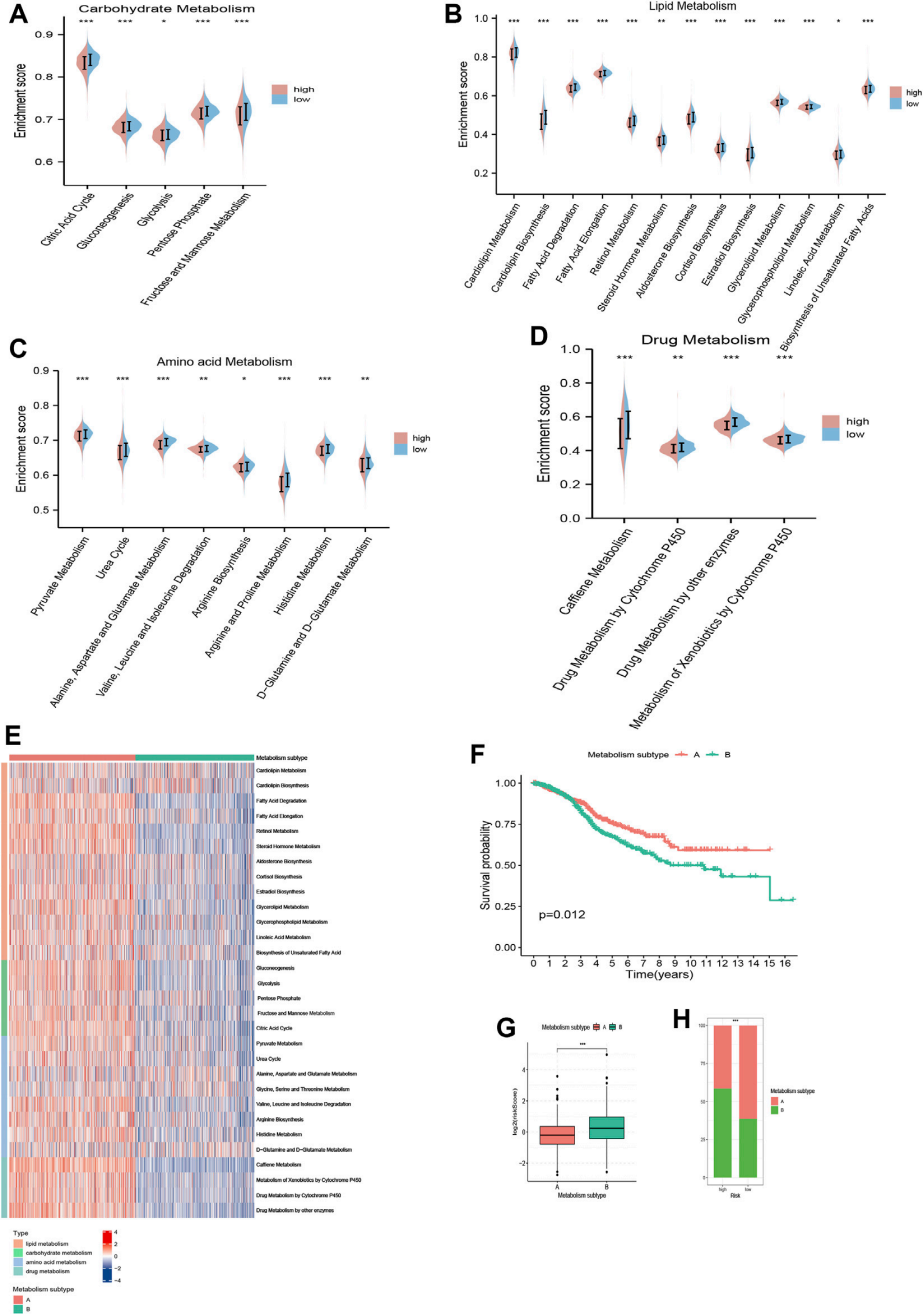
Metabolism analysis of TRMRG risk score signature. Violin plots comparing the metabolic activities of **(A)** carbohydrate, **(B)** lipid, **(C)** amino acid and **(D)** drug between high and low risk patients. **(E)** Distinctions in metabolic activities between two metabolism subtypes. **(F)** Kaplan-Meier plot for patients with metabolism subtype A and B. **(G)** Risk score differences between patients with metabolism subtype A and B. **(H)** Frequencies of two metabolism subtypes in high and low risk patients. Statistical Significance: *p < 0.05; **p < 0.01; ***p < 0.001; ns, not significant.

A total of 30 metabolism-related gene signatures were enrolled in the study, including 13 for lipid, 5 for carbohydrate, 8 for amino acid and 4 for drug metabolism. Overall, the metabolic activities for nutrients and drugs were stronger in low risk patients (Figures 8A–D). Furthermore, two metabolic subtypes were identified for CRC patients using consensus clustering method. As shown in Figure 8E, the overall metabolic activity was higher in metabolism subtype A than in subtype B. Kaplan-Meier analysis correlated metabolism subtype A with better OS (Figure 8F). The combinatorial analysis of metabolism subtypes and risk score signature suggested that patients with metabolism subtype B were associated with higher risk scores (Figures 8G, H). Therefore, our analysis above identified the diversified metabolic landscape as another contributor to disparities in prognosis between high and low risk patients, in addition to TME heterogeneity.

### Correlation of TRMRG risk score signature with sensitivity to anti-tumor drugs

**FIGURE 9:**
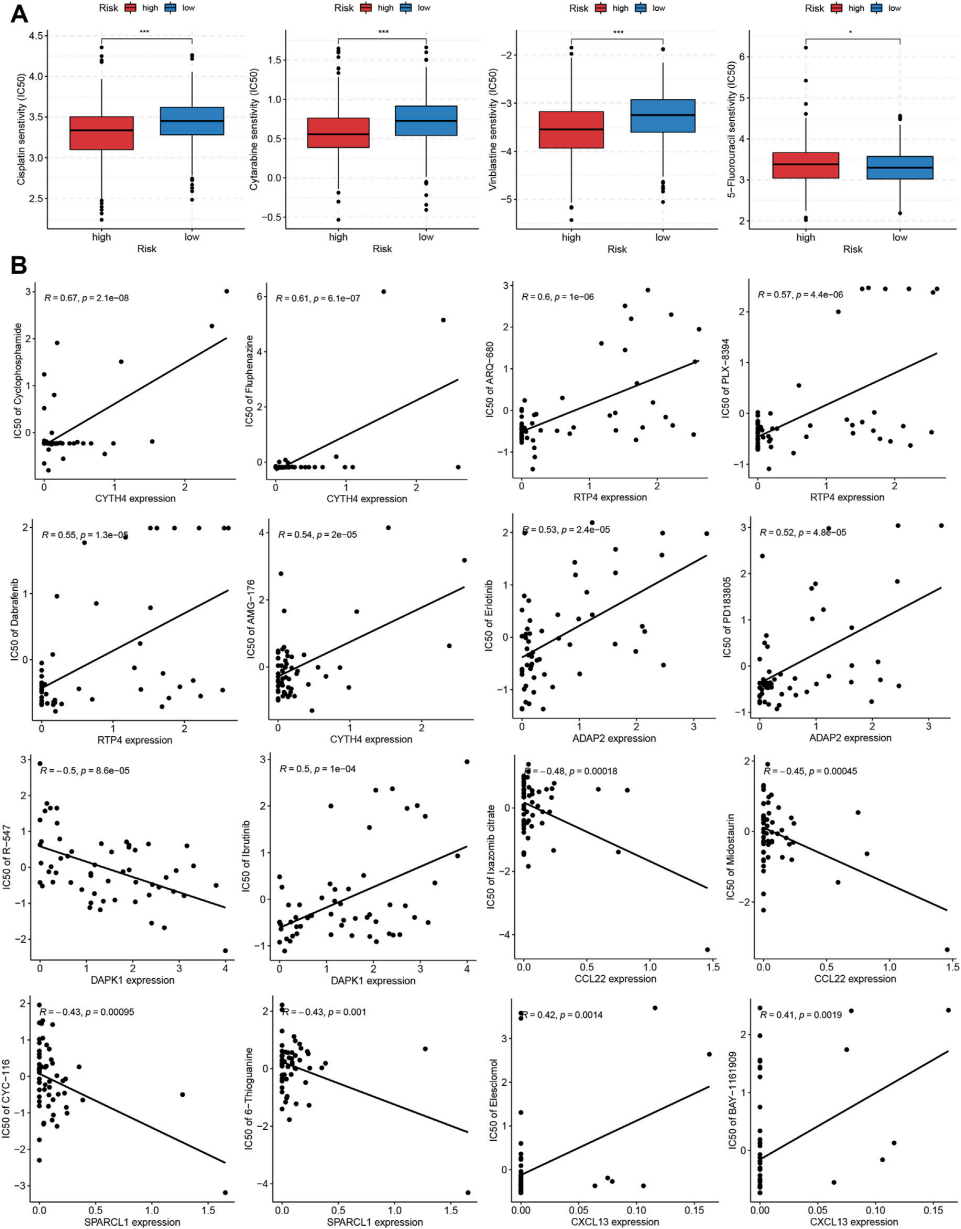
Correlation of TRMRG risk score signature with sensitivity to anti-tumor drugs. **(A)** Differences in IC50 values for cisplatin, cytarabine, vinblastine and 5-fluorouracil between high and low risk patients. **(B)** Pearson correlation analysis of TRMRG expression levels with IC50 values for anti-tumor drugs in NCI-60 cell lines. Statistical Significance: *p < 0.05; ***p < 0.001.

To evaluate the potential of TRMRG risk score in guiding clinical medication, we aimed to select the appropriate anti-tumor drugs for patients with high and low risk, respectively. It was suggested that high risk patients exhibited lower IC50 values for cisplatin, cytarabine, and vinblastine while low risk patients showed higher sensitivity to 5-fluorouracil (Figure 9A). Moreover, drug sensitivity profiles for NCI-60 cell line were downloaded from cell miner database and used to investigate the association between individual TRMRG expression and drug sensitivity. As shown in Figure 9B, the expression of CYTH4 was positively correlated with IC50s of cyclophosphamide, fluphenazine and AMG-176, suggesting that patients with high CYTH4 expression were likely to develop resistance to these three drugs. Likewise, up-regulated RTP4 expression was suggestive of impaired therapeutic efficacy of ARQ-680, PLX-8394 and dabrafenib. Positive associations between ADAP2 expression and resistance to erlotinib, PD183805, as well as between CXCL13 expression and resistance to elesclomol and BAY-1161909 were also observed. Increased DAPK1 expression indicated higher sensitivity to R-547 on the one hand, while indicated resistance to ibrutinib on the other hand. There were inverse associations between CCL22 expression and IC50s for ixazomib citrate and midostaurin. Similarly, improved therapeutic outcomes may also be achieved for patients with enhanced SPARCL1 expression after receiving CYC-116 and 6-thioguanine treatment.

### External validation of TRMRG risk score signature in predicting prognosis and therapeutic responses

To investigate the generality of TRMRG risk score signature, another two CRC cohorts: GSE17536 and GSE17537 were obtained from GEO database for external validation. In both of the two cohorts, high risk patients were characterized by significantly shortened OS (Supplementary Figures S12A, B). Time-dependent ROC analysis also verified the efficacy of the risk score in predicting OS (Supplementary Figures S12C, D). In GSE17536, patients with high risk exhibited significantly higher TIDE scores (*p* = 7.9e^−08^, Supplementary Figure S12E). While in GSE17537, increased TIDE scores with borderline significance (*p* = 0.052) could also be observed for high risk patients (Supplementary Figure S12F). In addition, non-responders to ICI therapies accounted for a larger portion in both of the two cohorts (Supplementary Figures S12G, H). Overall, the external validation process corroborated the stability and generality of TRMRG risk score signature in predicting survival and therapeutic responses for CRC patients.

### Development of a nomogram based on TRMRG risk score signature

**FIGURE 10:**
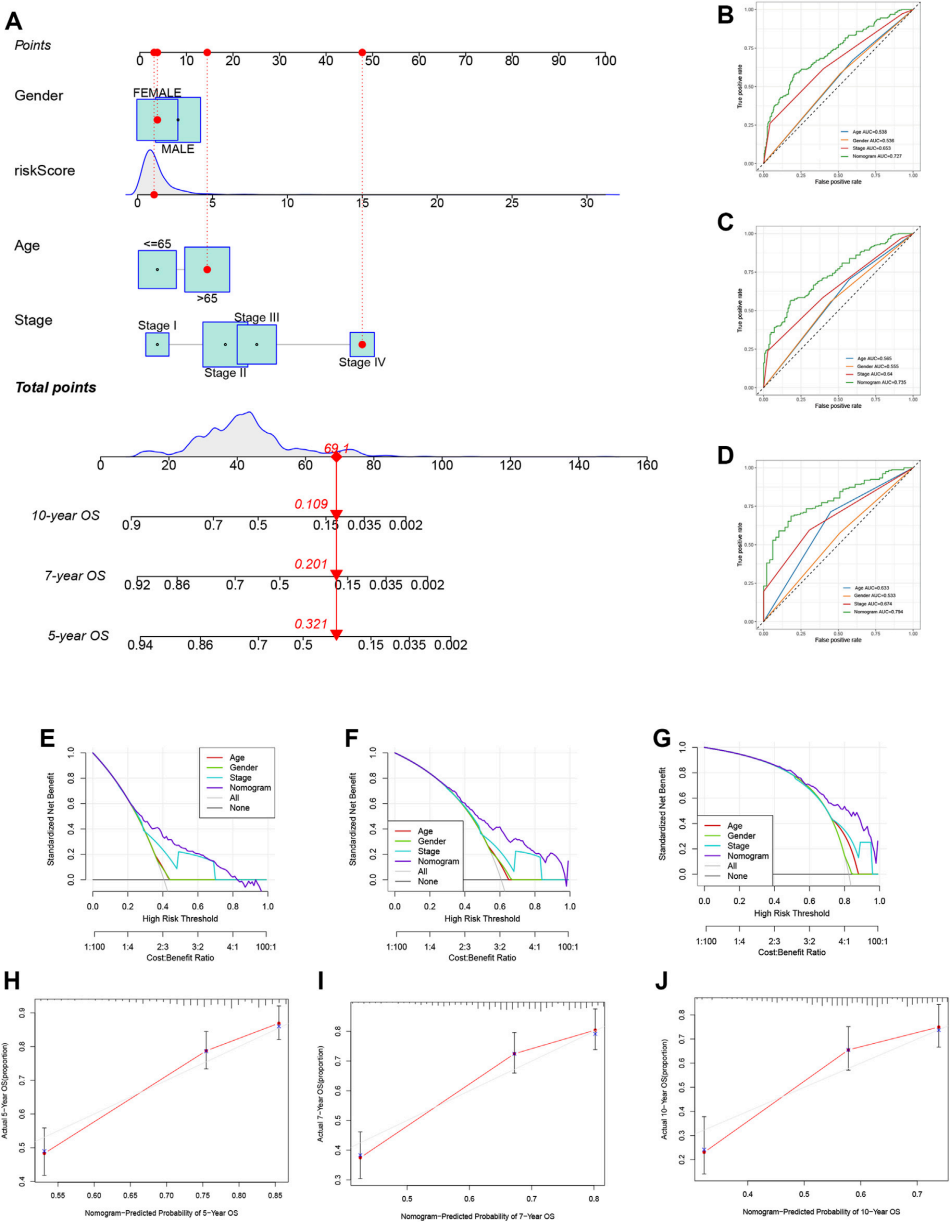
Development of a nomogram based on TRMRG risk score signature. **(A)** Nomogram integrating TRMRG risk score, age, gender and tumor stage for predicting 5, 7, 10 years OS. **(B–D)** Time-dependent ROC curves evaluating the efficacy of the total nomogram score in predicting **(B)** 5, **(C)** 7, **(D)** 10 years OS, in comparison to age, gender and tumor stage. **(E–G)** DCA curves estimating the efficacy of the nomogram in predicting **(E)** 5, **(F)** 7, **(G)**10 years OS from the perspective of clinical benefit. **(H–J)** Calibration curves of the nomogram for predicting **(H)** 5, **(I)** 7, **(J)**10 years OS. AUC: area under curve.

To further enhance the clinical application value of our TRMRG risk score signature, a nomogram integrating risk score, age, gender and tumor stage was developed in the meta-cohort. After endowing a value to each variable, a specific score was obtained. The total nomogram score could be estimated by adding up scores for each variable together (Figure 10A). The AUCs for predicting patients’ 5, 7 and 10 years OS were all over 0.700. The prediction of OS based on the total nomogram score showed higher accuracy than the method using age, gender and stage only (Figures 10B–D). In addition, DCA analysis revealed that the aggregate nomogram score displayed better net benefit than the age-, gender- and stage-only model for predicting time-dependent survival (Figures 10E–G). Besides, according to Figures 10H–J, calibration plots for 5-, 7- and 10-year OS showed good consistency between actual observations and predicted value of nomogram, which further highlighted the clinical application value of the nomogram in prognosis prediction.

## Discussion

With rapid development, scRNA-seq technology provides an unprecedented opportunity for us to unravel the molecular characteristics of diversified cell types infiltrated in TME, and opens up a new era of cancer research. With the help of this technology, researchers are unveiling the mystery of the phenotypical and functional variations of tumor infiltrating immune cells that contribute to heterogeneity of TME and different responses to immunotherapy. By analyzing scRNA-seq data obtained from small cell lung cancer patients, Tian et al. (2022) recognized multiple T Cell clusters with markedly different immune checkpoint gene expression patterns, which provided rationale for the application of personalized ICI strategies to different patient groups. A generally immunosuppressive TME characterized by abundant Treg infiltration and the absence of exhausted CD8 T cells for gastric cancer (GC) patients was identified through the scRNA-seq analysis performed by Li Y. et al. (2022), which to some extent accounted for GC patients’ low response rate towards ICI treatment. Notably, in an article published recently, Song et al. (2022) performed combinatorial analysis of single cell and bulk RNA-seq profiles and acquired some promising findings. First they selected marker genes of NK cells by analyzing scRNA-seq profiles derived from lung cancer patients, and latter applied the relevant genes to bulk RNA-seq profiles of large patient cohorts to construct a risk score model. The risk score model was corroborated to offer accurate prediction for the prognosis and immunotherapeutic response for lung cancer patients. Inspired by this, we attempted to investigate whether a prognostic model of this kind could be applied to CRC patients.

Trm is a newly-identified type of memory CD8 T cells lacking in migratory ability and encompasses tissue residency tendency. Previous studies have correlated the high infiltration level of Trm with favorable prognosis in several types of malignancies. However, there is still a lack of studies exploring the unique marker genes for Trm. Besides, the specific function of Trm in CRC patients remains to be fully elucidated. In the present study, Trm marker genes were obtained through the analysis of a single T cell RNA-seq dataset for CRC patients. Then, the marker genes obtained above were applied for consensus clustering analysis which classify CRC patients from TCGA and GEO cohorts into two clusters. Patients with Trm cluster A were characterized by enhanced immune cell infiltration and elevated immune activities. While patients with Trm cluster B exhibited increased tumor purity with few immune and stromal contents in TME. Furthermore, to find out more genes in relation to Trm, we defined TRMRGs as DEGs between two Trm clusters, which were used to develop a risk score signature to quantify patients risk level. High risk patients were associated with attenuated immunogenicity, weakened sensitivity to immunotherapy, as well as adverse clinical outcomes. While low risk patients with advantages in survival exhibited increased immunogenicity, stronger metabolic activity and higher sensitivity to immunotherapies.

Immunotherapy targeting at immune checkpoint genes, namely the ICI treatment has been gradually applied for clinical practice and demonstrated perfect therapeutic effect. In the context of CRC, monoclonal antibodies targeting at PD-1 such as nivolumab (Overman et al., 2017) and CTLA4 such as ipilimumab (Overman et al., 2018), exhibited high efficacy for the treatment of CRC patients with high microsatellite instability status. However, therapeutic sensitivity of ICI treatment is dependent on numerous factors, among which the heterogeneity in TME landscapes is an essential determinant. In our current study, low risk patients were characterized by higher infiltration levels of activated CD4 T cells, CD8 T cells and B cells with anti-tumor potency. Besides, the anti-cancer immunity in low risk patients were more easily to be primed, activated, and trafficked to tumor cells, and were more competent in recognizing and killing tumor cells. In addition, our analysis correlated low risk patients with higher immunogenicity as demonstrated by increased expressions of HLA family genes. Moreover, low risk patients encompassed elevated TCR richness and diversity. The expansion or alteration of TCR repertoires followed by ICI treatment has been observed by an increasing number of studies (Crosby et al., 2018; Yost et al., 2019; Someya et al., 2022), suggesting that ICI therapies rely heavily on the recruitment of new T Cells (Chen Y. et al, 2022). In fact, TCR has been identified as an indicator for predicting immunotherapeutic responses, with its low richness and diversity representing impaired sensitivity (Postow et al., 2015; Chen Y. et al, 2022). By contrast, for high risk patients, the richness of multiple immunosuppressive cells including MDSC, Tregs, Th2 cells, immature DC and the expression of immunosuppressive molecules including VEGFA and TGFβ was significantly higher in high risk patients as compared to low risk counterparts. MDSCs and Tregs are acknowledged as two “bad guys” with pro-tumorigenic and immunosuppressive properties in TME, of which the high infiltration levels represent impaired ICI therapeutic responses (Tay et al., 2020; De Sanctis et al., 2022). Mechanistically, Th2 cells mediate the progression of CRC *via* the secretion of some carcinogenic cytokines, indicating the potential therapeutic utility of Th2 cells as novel targets for cancer immunotherapy (Akimoto and Takenaga, 2019; Knudson et al., 2022). Previous studies also identified immature DCs (Song et al., 2018) and inhibitory molecules including VEGFA (Terme et al., 2013) and TGF-β (Liu et al., 2022) to be negative regulators for ICI therapeutic response. In addition, increased stromal contents and elevated stromal pathways were also associated with high risk patients, which may hinder the immune cells from entering into the tumor site and undermine the anti-tumor responses (Salmon et al., 2012). Based on the above analysis, it was concluded that high risk patients encompassed an immunosuppressive TME, which was responsible for impaired sensitivity to ICI treatment. In support of this, high risk patients were associated with decreased IPS scores for anti-PD1 and/or anti CTLA4 treatment, as well as elevated TIDE score. Moreover, the application of TRMRG model to two immunotherapeutic cohort yielded identical results: high risk patients exhibited either impaired response rate or shortened OS after receiving ICI treatment.

Two chemokine-encoding genes: CCL22 and CXCL13, were involved in the present TRMRG risk score signature. Through the interaction with CCR4, CCL22 could facilitate the recruitment of immunosuppressive cells (Yoshie, 2021). Moreover, elegant experiments have provided evidence for CCL22’s oncogenic role in multiple types of cancers: CCL22 overexpression renders an immunosuppressive TME and is associated shortened OS for cervical cancer patients (Ni et al., 2022); the intimate association between CCL22 and disease progression was revealed in esophageal cancer patients (Chen J. et al, 2022). By contrast, the specific role of CXCL13 in TME was more complex. On the one hand, it was revealed by some studies that up-regulated CXCL13 expression could promote the proliferation, migration and invasion of CRC cells (Zhu et al., 2015), and was responsible for the dismal survival and drug resistance for CRC patients (Qi et al., 2014; Zhang et al., 2020). On the other hand, CXCL13 was also reported to be fully capable of attracting anti-tumor immune cells to TME and correlated with favorable prognosis (Bindea et al., 2013). Therefore, a deeper understanding about the potential role of CXCL13 in TME remodeling is crucial for unraveling the dual effect of the gene in tumorigenesis.

To augment the association between TRMRG risk score signature and clinical practice, we sought to investigate the suitable therapeutic strategies for patients at different risk levels. It was revealed that high risk patients demonstrated higher sensitivity to a wider range of anti-tumor drugs, including cisplatin, cytarabine and vinblastin. While low risk patients responded better to 5-fluorouracil. Finally, a nomogram integrating TRMRG risk score, age, gender and tumor stage was established to enhance the clinical utility of our work.

Admittedly, the present study had certain limitations. First, although the identification of Trm marker genes were achieved by dissecting a single T Cell transcriptome dataset, the supplementation of newly-emerged Trm markers is still necessary. In addition, as a descriptive research performed by retrospectively analyzing public databases, our current work lacks validation in the real world. Therefore, future prospective and large-scale researches are warranted to verify the current findings.

## Conclusion

In summary, through integrative analysis of single cell and bulk RNA transcriptomic profiles, the present study identified two Trm clusters with significantly different TME landscapes for CRC patients, which highlighted the non-negligible role of Trm in regulating the complexity and heterogeneity of TME. Moreover, we quantified the risk level based on Trm-related genes and established a seven-gene risk score signature. The potential utility of the signature to serve as a novel clinical biomarker for prediction of the prognosis and immunotherapeutic responses was also corroborated.

## Data Availability

The datasets presented in this study can be found in online repositories. The names of the repository/repositories and accession number(s) can be found in the article/Supplementary Material.

## Abbreviations

CRC, colorectal cancer; TRMRG, tissue memory CD8 T cell-related gene; TCGA, the cancer genome atlas; COAD, colon adenocarcinoma; READ, rectal adenocarcinoma; LASSO, least absolute shrinkage and selection operator; AUC, area under the curve; KEGG, Kyoto encyclopedia of genes and genomes; OS, overall survival; GSEA, gene set enrichment analysis; ssGSEA, single sample gene set enrichment analysis; IC50, half inhibitory concentration; GO, gene ontology; TME, tumor microenvironment; TIDE, tumor immune dysfunction and exclusion; TCIA, the cancer immunome atlas; GEO, gene expression omnibus.

## References

[B1] Akimoto M. Takenaga K. (2019). Role of the IL-33/ST2L axis in colorectal cancer progression. Cell. Immunol. 343, 103740. 10.1016/j.cellimm.2017.12.014 29329638

[B2] Bindea G. Mlecnik B. Tosolini M. Kirilovsky A. Waldner M. Obenauf A. C. (2013). Spatiotemporal dynamics of intratumoral immune cells reveal the immune landscape in human cancer. Immunity 39 (4), 782–795. 10.1016/j.immuni.2013.10.003 24138885

[B3] Boyman O. Hefti H. P. Conrad C. Nickoloff B. J. Suter M. Nestle F. O. (2004). Spontaneous development of psoriasis in a new animal model shows an essential role for resident T cells and tumor necrosis factor-alpha. J. Exp. Med. 199 (5), 731–736. 10.1084/jem.20031482 14981113 PMC2213300

[B4] Charoentong P. Finotello F. Angelova M. Mayer C. Efremova M. Rieder D. (2017). Pan-cancer immunogenomic analyses reveal genotype-immunophenotype relationships and predictors of response to checkpoint blockade. Cell. Rep. 18 (1), 248–262. 10.1016/j.celrep.2016.12.019 28052254

[B5] Chen D. S. Mellman I. (2013). Oncology meets immunology: The cancer-immunity cycle. Immunity 39 (1), 1–10. 10.1016/j.immuni.2013.07.012 23890059

[B6] Chen J. Zhao D. Zhang L. Zhang J. Xiao Y. Wu Q. (2022a). Tumor-associated macrophage (TAM)-derived CCL22 induces FAK addiction in esophageal squamous cell carcinoma (ESCC). Cell. Mol. Immunol. 19 (9), 1054–1066. 10.1038/s41423-022-00903-z 35962191 PMC9424285

[B7] Chen Y. Bai B. Ying K. Pan H. Xie B. (2022b). Anti-PD-1 combined with targeted therapy: Theory and practice in gastric and colorectal cancer. Biochim. Biophys. Acta Rev. Cancer 1877 (5), 188775. 10.1016/j.bbcan.2022.188775 35934154

[B8] Clarke J. Panwar B. Madrigal A. Singh D. Gujar R. Wood O. (2019). Single-cell transcriptomic analysis of tissue-resident memory T cells in human lung cancer. J. Exp. Med. 216 (9), 2128–2149. 10.1084/jem.20190249 31227543 PMC6719422

[B9] Conesa A. Madrigal P. Tarazona S. Gomez-Cabrero D. Cervera A. McPherson A. (2016). A survey of best practices for RNA-seq data analysis. Genome Biol. 17, 13. 10.1186/s13059-016-0881-8 26813401 PMC4728800

[B10] Crosby E. J. Wei J. Yang X. Y. Lei G. Wang T. Liu C. X. (2018). Complimentary mechanisms of dual checkpoint blockade expand unique T-cell repertoires and activate adaptive anti-tumor immunity in triple-negative breast tumors. Oncoimmunology 7 (5), e1421891. 10.1080/2162402X.2017.1421891 29721371 PMC5927534

[B11] de Miguel M. Calvo E. (2020). Clinical challenges of immune checkpoint inhibitors. Cancer Cell. 38 (3), 326–333. 10.1016/j.ccell.2020.07.004 32750319

[B12] De Sanctis F. Adamo A. Cane S. Ugel S. (2022). Targeting tumour-reprogrammed myeloid cells: The new battleground in cancer immunotherapy. Semin. Immunopathol., 1–24. 10.1007/s00281-022-00965-1 PMC9513014 36161514

[B13] Du K. Zou J. Wang B. Liu C. Khan M. Xie T. (2022). A metabolism-related gene prognostic index bridging metabolic signatures and antitumor immune cycling in head and neck squamous cell carcinoma. Front. Immunol. 13, 857934. 10.3389/fimmu.2022.857934 35844514 PMC9282908

[B14] Duan Q. Zhang H. Zheng J. Zhang L. (2020). Turning cold into hot: Firing up the tumor microenvironment. Trends Cancer 6 (7), 605–618. 10.1016/j.trecan.2020.02.022 32610070

[B15] Edwards J. Wilmott J. S. Madore J. Gide T. N. Quek C. Tasker A. (2018). CD103(+) tumor-resident CD8(+) T cells are associated with improved survival in immunotherapy-naive melanoma patients and expand significantly during anti-PD-1 treatment. Clin. Cancer Res. 24 (13), 3036–3045. 10.1158/1078-0432.CCR-17-2257 29599411

[B16] Gao J. Zhao Z. Zhang H. Huang S. Xu M. Pan H. (2022). Transcriptomic characterization and construction of M2 macrophage-related prognostic and immunotherapeutic signature in ovarian metastasis of gastric cancer. Cancer Immunol Immunother. 10.1007/s00262-022-03316-z PMC10991875 36336725

[B17] Guo J. N. Chen D. Deng S. H. Huang J. R. Song J. X. Li X. Y. (2022). Identification and quantification of immune infiltration landscape on therapy and prognosis in left- and right-sided colon cancer. Cancer Immunol. Immunother. 71 (6), 1313–1330. 10.1007/s00262-021-03076-2 34657172 PMC9122887

[B18] Jiang P. Gu S. Pan D. Fu J. Sahu A. Hu X. (2018). Signatures of T cell dysfunction and exclusion predict cancer immunotherapy response. Nat. Med. 24 (10), 1550–1558. 10.1038/s41591-018-0136-1 30127393 PMC6487502

[B19] Jiang Q. Chen H. Tang Z. Sun J. Ruan Y. Liu F. (2021). Stemness-related LncRNA pair signature for predicting therapy response in gastric cancer. BMC Cancer 21 (1), 1067. 10.1186/s12885-021-08798-1 34587919 PMC8482617

[B20] Kim H. D. Jeong S. Park S. Lee Y. J. Ju Y. S. Kim D. (2021). Implication of CD69(+) CD103(+) tissue-resident-like CD8(+) T cells as a potential immunotherapeutic target for cholangiocarcinoma. Liver Int. 41 (4), 764–776. 10.1111/liv.14814 33548061

[B21] Kim S. K. Schluns K. S. Lefrancois L. (1999). Induction and visualization of mucosal memory CD8 T cells following systemic virus infection. J. Immunol. 163 (8), 4125 10510347

[B22] Knudson K. M. Hwang S. McCann M. S. Joshi B. H. Husain S. R. Puri R. K. (2022). Recent advances in IL-13rα2-directed cancer immunotherapy. Front. Immunol. 13, 878365. 10.3389/fimmu.2022.878365 35464460 PMC9023787

[B23] Li X. Wang R. Wang S. Wang L. Yu J. (2022a). Construction of a B cell-related gene pairs signature for predicting prognosis and immunotherapeutic response in non-small cell lung cancer. Front. Immunol. 13, 989968. 10.3389/fimmu.2022.989968 36389757 PMC9647047

[B24] Li Y. Hu X. Lin R. Zhou G. Zhao L. Zhao D. (2022b). Single-cell landscape reveals active cell subtypes and their interaction in the tumor microenvironment of gastric cancer. Theranostics 12 (8), 3818–3833. 10.7150/thno.71833 35664061 PMC9131288

[B25] Liu J. Tao H. Yuan T. Li J. Li J. Liang H. (2022). Immunomodulatory effects of regorafenib: Enhancing the efficacy of anti-PD-1/PD-L1 therapy. Front. Immunol. 13, 992611. 10.3389/fimmu.2022.992611 36119072 PMC9479218

[B26] Mariathasan S. Turley S. J. Nickles D. Castiglioni A. Yuen K. Wang Y. (2018). TGFβ attenuates tumour response to PD-L1 blockade by contributing to exclusion of T cells. Nature 554 (7693), 544–548. 10.1038/nature25501 29443960 PMC6028240

[B27] Ni H. Zhang H. Li L. Huang H. Guo H. Zhang L. (2022). T cell-intrinsic STING signaling promotes regulatory T cell induction and immunosuppression by upregulating FOXP3 transcription in cervical cancer. J. Immunother. Cancer 10 (9), e005151. 10.1136/jitc-2022-005151 36126994 PMC9490630

[B28] Overman M. J. Lonardi S. Wong K. Y. M. Lenz H. J. Gelsomino F. Aglietta M. (2018). Durable clinical benefit with nivolumab plus ipilimumab in DNA mismatch repair-deficient/microsatellite instability-high metastatic colorectal cancer. J. Clin. Oncol. 36 (8), 773–779. 10.1200/JCO.2017.76.9901 29355075

[B29] Overman M. J. McDermott R. Leach J. L. Lonardi S. Lenz H.-J. Morse M. A. (2017). Nivolumab in patients with metastatic DNA mismatch repair-deficient or microsatellite instability-high colorectal cancer (CheckMate 142): An open-label, multicentre, phase 2 study. Lancet Oncol. 18 (9), 1182–1191. 10.1016/S1470-2045(17)30422-9 28734759 PMC6207072

[B30] Pardoll D. M. (2012). The blockade of immune checkpoints in cancer immunotherapy. Nat. Rev. Cancer 12 (4), 252–264. 10.1038/nrc3239 22437870 PMC4856023

[B31] Park S. L. Mackay L. K. Waithman J. Gebhardt T. (2019). Tissue-resident memory T cells orchestrate tumour-immune equilibrium. Cell. Stress 3 (5), 162–164. 10.15698/cst2019.05.187 31225511 PMC6551858

[B32] Pitt J. M. Marabelle A. Eggermont A. Soria J. C. Kroemer G. Zitvogel L. (2016). Targeting the tumor microenvironment: Removing obstruction to anticancer immune responses and immunotherapy. Ann. Oncol. 27 (8), 1482–1492. 10.1093/annonc/mdw168 27069014

[B33] Postow M. A. Manuel M. Wong P. Yuan J. Dong Z. Liu C. (2015). Peripheral T cell receptor diversity is associated with clinical outcomes following ipilimumab treatment in metastatic melanoma. J. Immunother. Cancer 3, 23. 10.1186/s40425-015-0070-4 26085931 PMC4469400

[B34] Qi X. W. Xia S. H. Yin Y. Jin L. F. Pu Y. Hua D. (2014). Expression features of CXCR5 and its ligand, CXCL13 associated with poor prognosis of advanced colorectal cancer. Eur. Rev. Med. Pharmacol. Sci. 18 (13), 1916–1924. 25010623

[B35] Reinhold W. C. Sunshine M. Liu H. Varma S. Kohn K. W. Morris J. (2012). CellMiner: A web-based suite of genomic and pharmacologic tools to explore transcript and drug patterns in the NCI-60 cell line set. Cancer Res. 72 (14), 3499–3511. 10.1158/0008-5472.CAN-12-1370 22802077 PMC3399763

[B36] Sallusto F. Lenig D. Forster R. Lipp M. Lanzavecchia A. (1999). Two subsets of memory T lymphocytes with distinct homing potentials and effector functions. Nature 401 (6754), 708–712. 10.1038/44385 10537110

[B37] Salmon H. Franciszkiewicz K. Damotte D. Dieu-Nosjean M. C. Validire P. Trautmann A. (2012). Matrix architecture defines the preferential localization and migration of T cells into the stroma of human lung tumors. J. Clin. Investig. 122 (3), 899–910. 10.1172/JCI45817 22293174 PMC3287213

[B38] Savas P. Virassamy B. Ye C. Salim A. Mintoff C. P. Caramia F. (2018). Single-cell profiling of breast cancer T cells reveals a tissue-resident memory subset associated with improved prognosis. Nat. Med. 24 (7), 986–993. 10.1038/s41591-018-0078-7 29942092

[B39] Sharma P. Hu-Lieskovan S. Wargo J. A. Ribas A. (2017). Primary, adaptive, and acquired resistance to cancer immunotherapy. Cell. 168 (4), 707–723. 10.1016/j.cell.2017.01.017 28187290 PMC5391692

[B40] Someya M. Tokita S. Kanaseki T. Kitagawa M. Hasegawa T. Tsuchiya T. (2022). Combined chemoradiotherapy and programmed cell death-ligand 1 blockade leads to changes in the circulating T-cell receptor repertoire of patients with non-small-cell lung cancer. Cancer Sci. 10.1111/cas.15566 PMC9746040 36069051

[B41] Song M. Chen X. Wang L. Zhang Y. (2018). Future of anti-PD-1/PD-L1 applications: Combinations with other therapeutic regimens. Chin. J. Cancer Res. 30 (2), 157–172. 10.21147/j.issn.1000-9604.2018.02.01 29861603 PMC5953954

[B42] Song P. Li W. Guo L. Ying J. Gao S. He J. (2022). Identification and validation of a novel signature based on NK cell marker genes to predict prognosis and immunotherapy response in lung adenocarcinoma by integrated analysis of single-cell and bulk RNA-sequencing. Front. Immunol. 13, 850745. 10.3389/fimmu.2022.850745 35757748 PMC9231585

[B43] Sung H. Ferlay J. Siegel R. L. Laversanne M. Soerjomataram I. Jemal A. (2021). Global cancer statistics 2020: GLOBOCAN estimates of incidence and mortality worldwide for 36 cancers in 185 countries. CA Cancer J. Clin. 71 (3), 209–249. 10.3322/caac.21660 33538338

[B44] Tay C. Qian Y. Sakaguchi S. (2020). Hyper-progressive disease: The potential role and consequences of T-regulatory cells foiling anti-PD-1 cancer immunotherapy. Cancers (Basel) 13 (1), 48. 10.3390/cancers13010048 33375291 PMC7796137

[B45] Terme M. Pernot S. Marcheteau E. Sandoval F. Benhamouda N. Colussi O. (2013). VEGFA-VEGFR pathway blockade inhibits tumor-induced regulatory T-cell proliferation in colorectal cancer. Cancer Res. 73 (2), 539–549. 10.1158/0008-5472.CAN-12-2325 23108136

[B46] Tian Y. Li Q. Yang Z. Zhang S. Xu J. Wang Z. (2022). Single-cell transcriptomic profiling reveals the tumor heterogeneity of small-cell lung cancer. Signal Transduct. Target Ther. 7 (1), 346. 10.1038/s41392-022-01150-4 36195615 PMC9532437

[B47] Wang H. Liu J. Yang J. Wang Z. Zhang Z. Peng J. (2022). A novel tumor mutational burden-based risk model predicts prognosis and correlates with immune infiltration in ovarian cancer. Front. Immunol. 13, 943389. 10.3389/fimmu.2022.943389 36003381 PMC9393426

[B48] Webb J. R. Milne K. Nelson B. H. (2015). PD-1 and CD103 are widely coexpressed on prognostically favorable intraepithelial CD8 T cells in human ovarian cancer. Cancer Immunol. Res. 3 (8), 926–935. 10.1158/2326-6066.CIR-14-0239 25957117

[B49] Wen P. Wang H. Li Y. Sui X. Hou Z. Guo X. (2022). MICALL2 as a substrate of ubiquitinase TRIM21 regulates tumorigenesis of colorectal cancer. Cell. Commun. Signal 20 (1), 170. 10.1186/s12964-022-00984-3 36307841 PMC9615392

[B50] Xu H. H. Wang H. L. Xing T. J. Wang X. Q. (2022). A novel prognostic risk model for cervical cancer based on immune checkpoint HLA-G-driven differentially expressed genes. Front. Immunol. 13, 851622. 10.3389/fimmu.2022.851622 35924232 PMC9341272

[B51] Xu L. Deng C. Pang B. Zhang X. Liu W. Liao G. (2018). Tip: A web server for resolving tumor immunophenotype profiling. Cancer Res. 78 (23), 6575–6580. 10.1158/0008-5472.CAN-18-0689 30154154

[B52] Xue W. Dong B. Zhao Y. Wang Y. Yang C. Xie Y. (2021). Upregulation of TTYH3 promotes epithelial-to-mesenchymal transition through Wnt/β-catenin signaling and inhibits apoptosis in cholangiocarcinoma. Cell. Oncol. (Dordr) 44 (6), 1351–1361. 10.1007/s13402-021-00642-9 34796468 PMC12980733

[B53] Yang C. Huang X. Liu Z. Qin W. Wang C. (2020). Metabolism-associated molecular classification of hepatocellular carcinoma. Mol. Oncol. 14 (4), 896–913. 10.1002/1878-0261.12639 31955511 PMC7138397

[B54] Yang W. Guo C. Herman J. G. Zhu C. Lv H. Su X. (2022). Epigenetic silencing of JAM3 promotes esophageal cancer development by activating Wnt signaling. Clin. Epigenetics 14 (1), 164. 10.1186/s13148-022-01388-3 36461092 PMC9719220

[B55] Yenyuwadee S. Sanchez-Trincado Lopez J. L. Shah R. Rosato P. C. Boussiotis V. A. (2022). The evolving role of tissue-resident memory T cells in infections and cancer. Sci. Adv. 8 (33), eabo5871. 10.1126/sciadv.abo5871 35977028 PMC9385156

[B56] Yoshie O. (2021). CCR4 as a therapeutic target for cancer immunotherapy. Cancers (Basel) 13 (21), 5542. 10.3390/cancers13215542 34771703 PMC8583476

[B57] Yoshihara K. Shahmoradgoli M. Martinez E. Vegesna R. Kim H. Torres-Garcia W. (2013). Inferring tumour purity and stromal and immune cell admixture from expression data. Nat. Commun. 4, 2612. 10.1038/ncomms3612 24113773 PMC3826632

[B58] Yost K. E. Satpathy A. T. Wells D. K. Qi Y. Wang C. Kageyama R. (2019). Clonal replacement of tumor-specific T cells following PD-1 blockade. Nat. Med. 25 (8), 1251–1259. 10.1038/s41591-019-0522-3 31359002 PMC6689255

[B59] Zhang G. Luo X. Zhang W. Chen E. Xu J. Wang F. (2020). CXCL-13 regulates resistance to 5-fluorouracil in colorectal cancer. Cancer Res. Treat. 52 (2), 622–633. 10.4143/crt.2019.593 32019285 PMC7176956

[B60] Zhang J. Wang H. Wu J. Yuan C. Chen S. Liu S. (2022). GALNT1 enhances malignant phenotype of gastric cancer via modulating CD44 glycosylation to activate the wnt/β-catenin signaling pathway. Int. J. Biol. Sci. 18 (16), 6068–6083. 10.7150/ijbs.73431 36439876 PMC9682532

[B61] Zhang L. Yu X. Zheng L. Zhang Y. Li Y. Fang Q. (2018). Lineage tracking reveals dynamic relationships of T cells in colorectal cancer. Nature 564 (7735), 268–272. 10.1038/s41586-018-0694-x 30479382

[B62] Zhu M. Zhang J. Bian S. Zhang X. Shen Y. Ni Z. (2022). Circadian gene CSNK1D promoted the progression of hepatocellular carcinoma by activating Wnt/β-catenin pathway via stabilizing Dishevelled Segment Polarity Protein 3. Biol. Proced. Online 24 (1), 21. 10.1186/s12575-022-00183-x 36460966 PMC9717411

[B63] Zhu Z. Zhang X. Guo H. Fu L. Pan G. Sun Y. (2015). CXCL13-CXCR5 axis promotes the growth and invasion of colon cancer cells via PI3K/AKT pathway. Mol. Cell. Biochem. 400 (1-2), 287–295. 10.1007/s11010-014-2285-y 25476740

