# Supplementary figures and images for: Identification of a tissue resident memory CD8 T cell-related risk score signature for colorectal cancer, the association with TME landscapes and therapeutic responses
Source: Front Genet. 2023 Jan 4;13:1088230. doi: 10.3389/fgene.2022.1088230 (PMC9845416; doi:10.3389/fgene.2022.1088230)

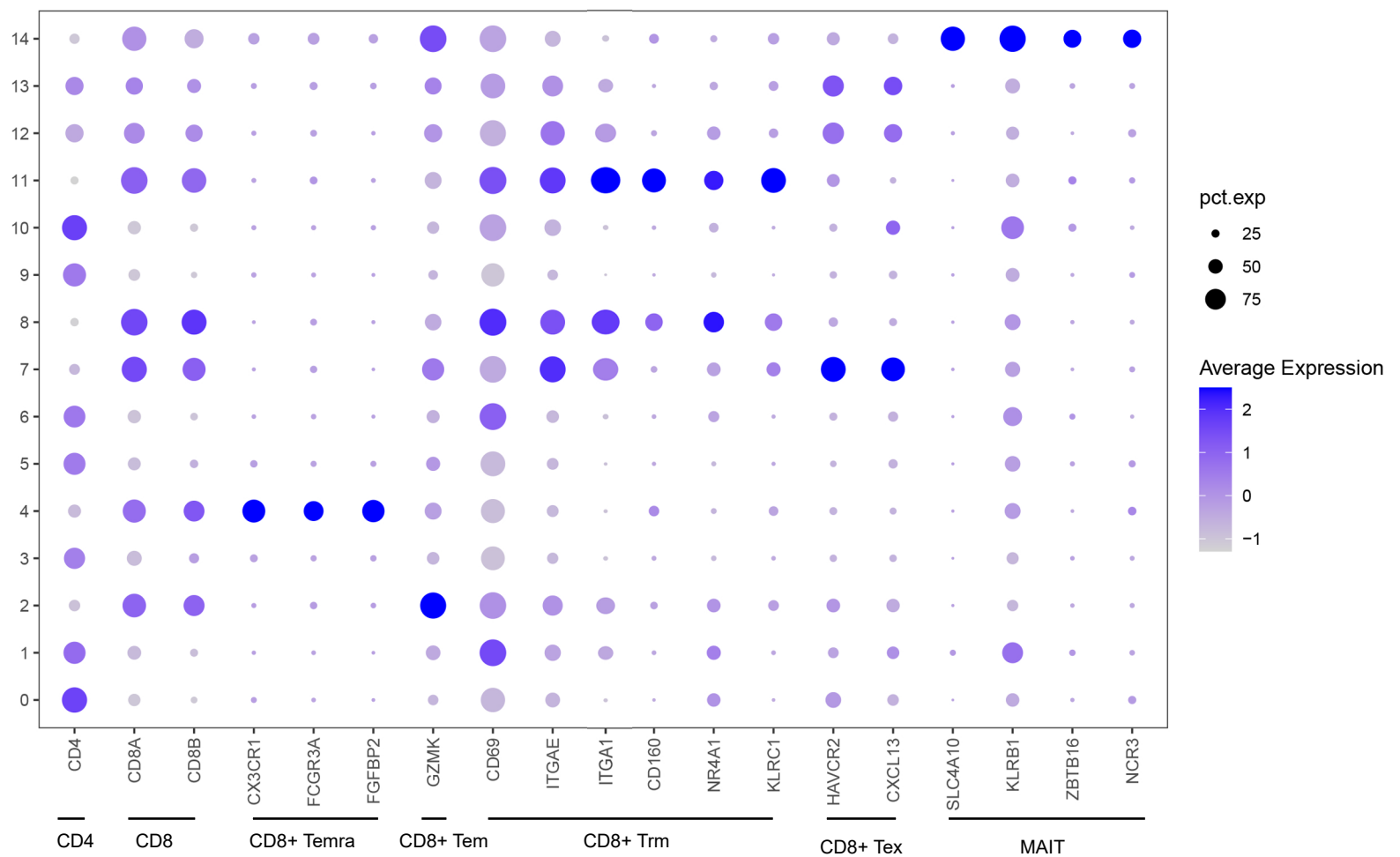

Supplement: Supplementary file 1 [file DataSheet2.PDF]

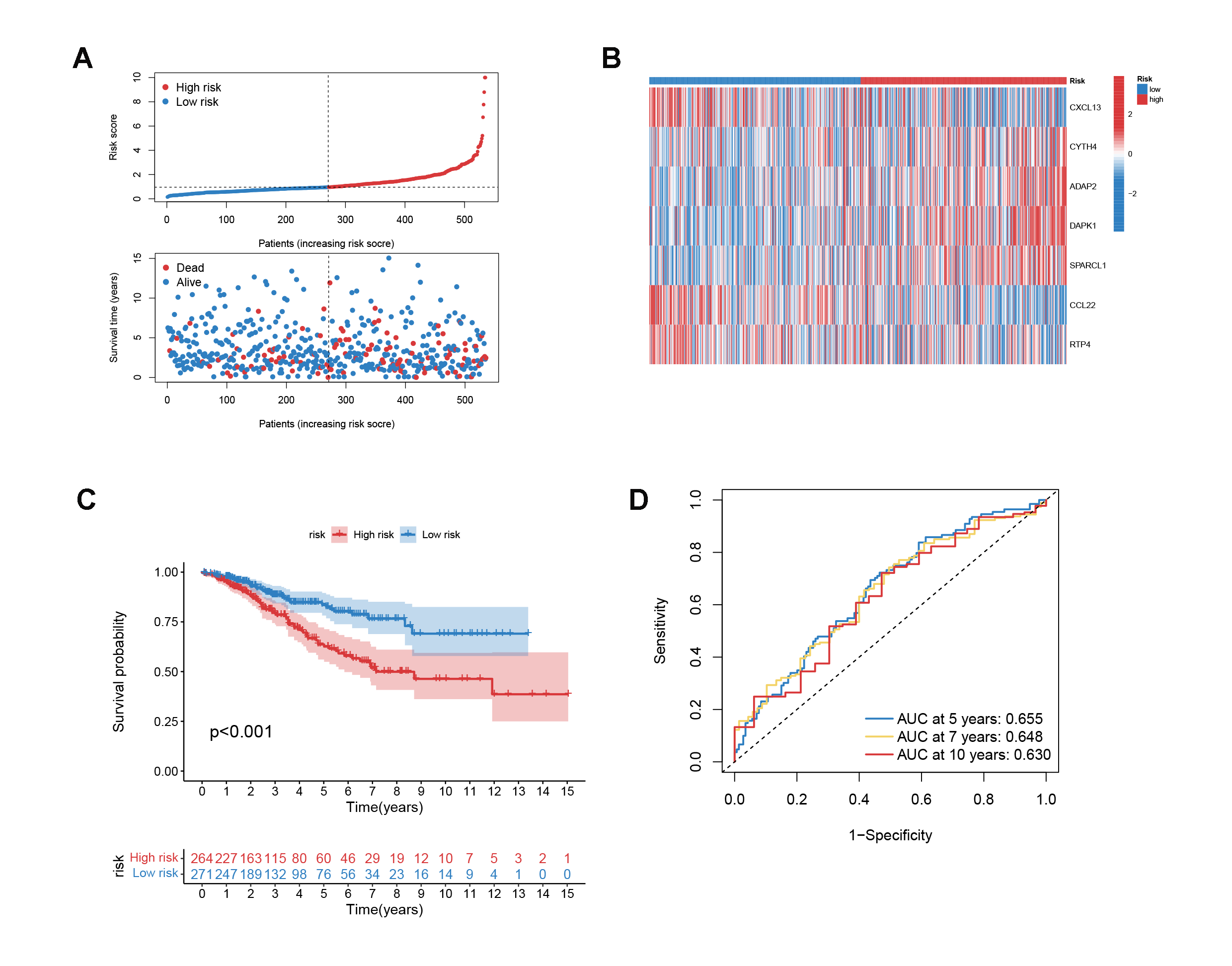

Supplement: Supplementary file 2 [file Image6.TIF]

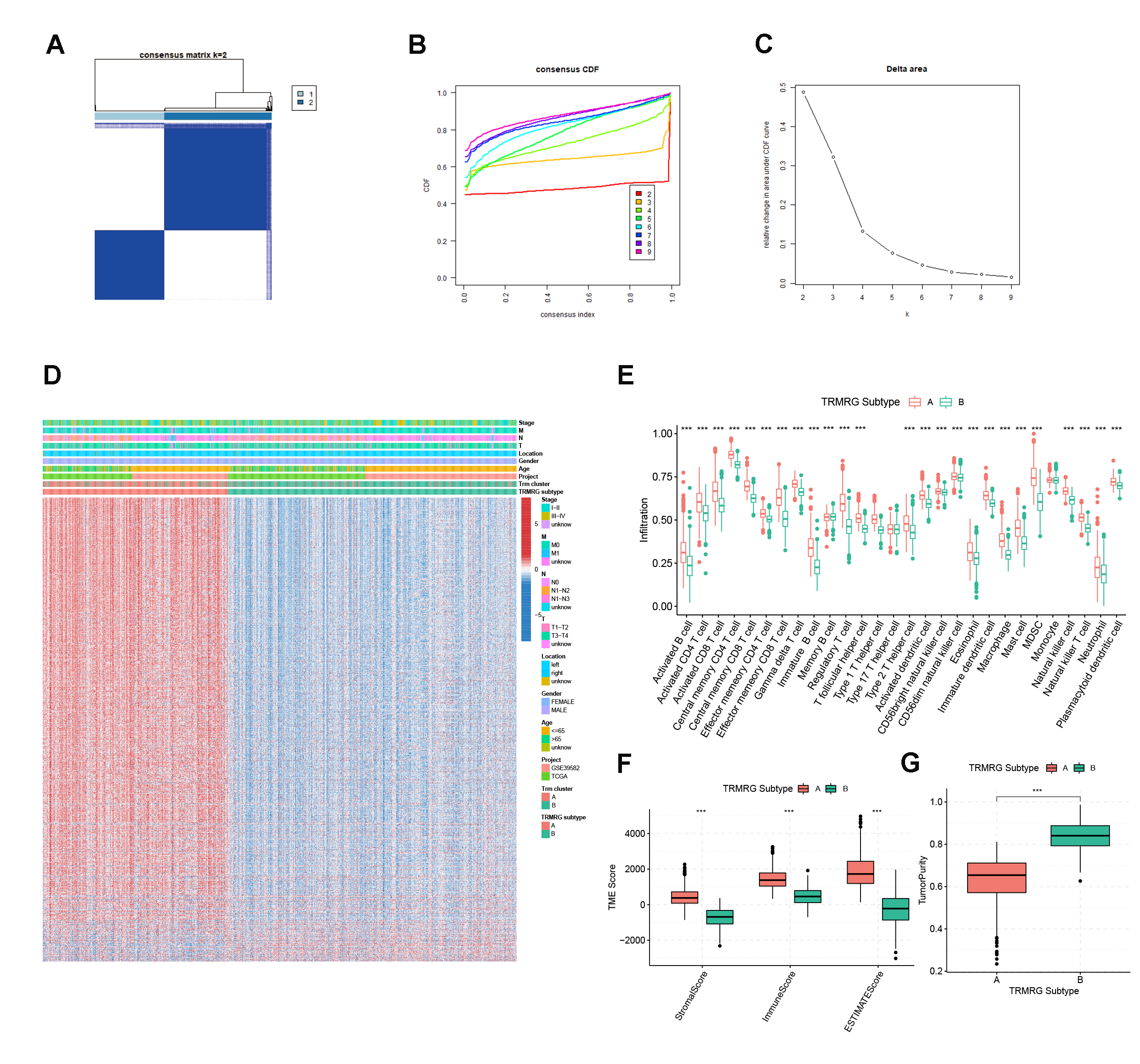

Supplement: Supplementary file 3 [file Image3.TIF]

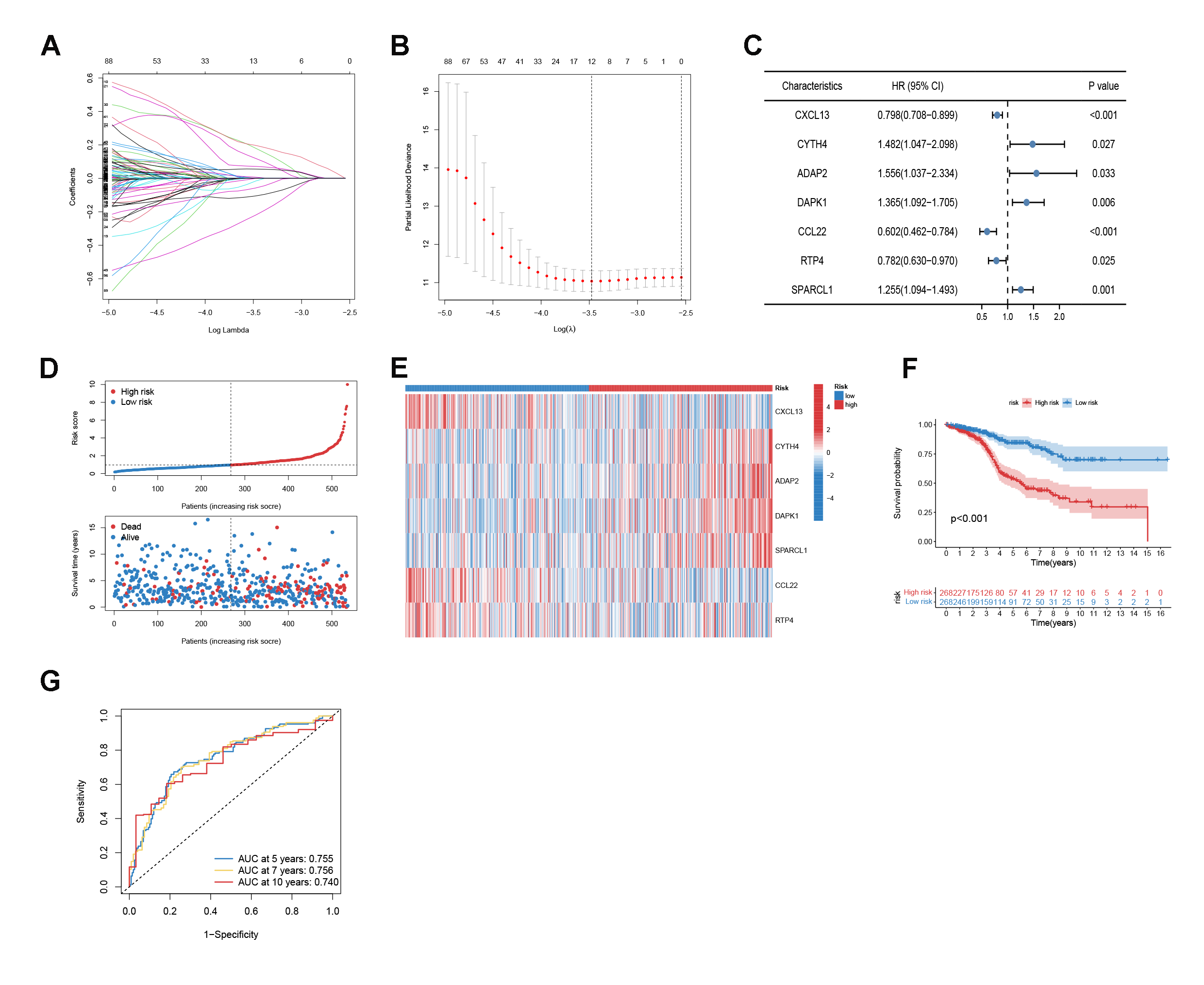

Supplement: Supplementary file 4 [file Image4.TIF]

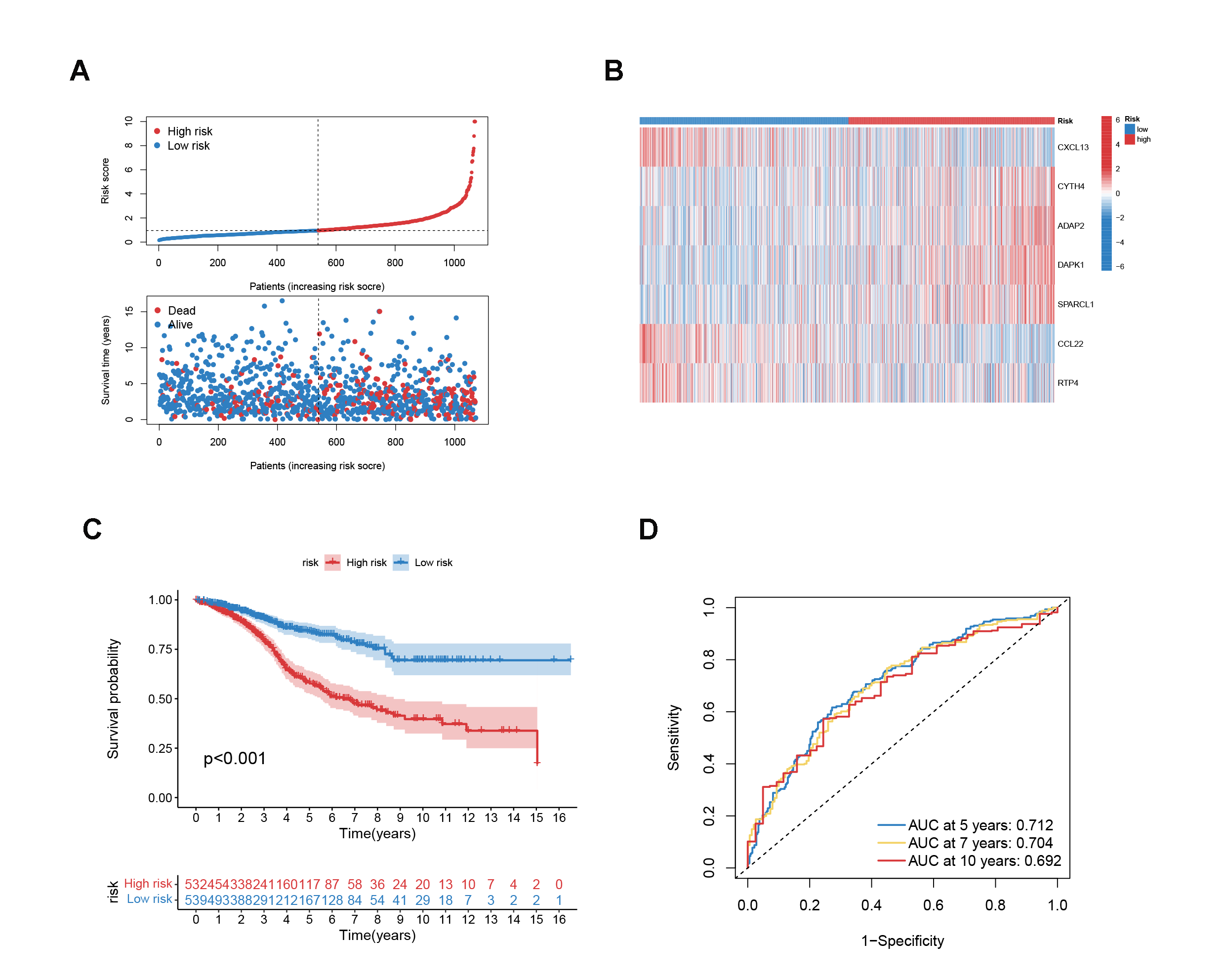

Supplement: Supplementary file 5 [file Image9.TIF]

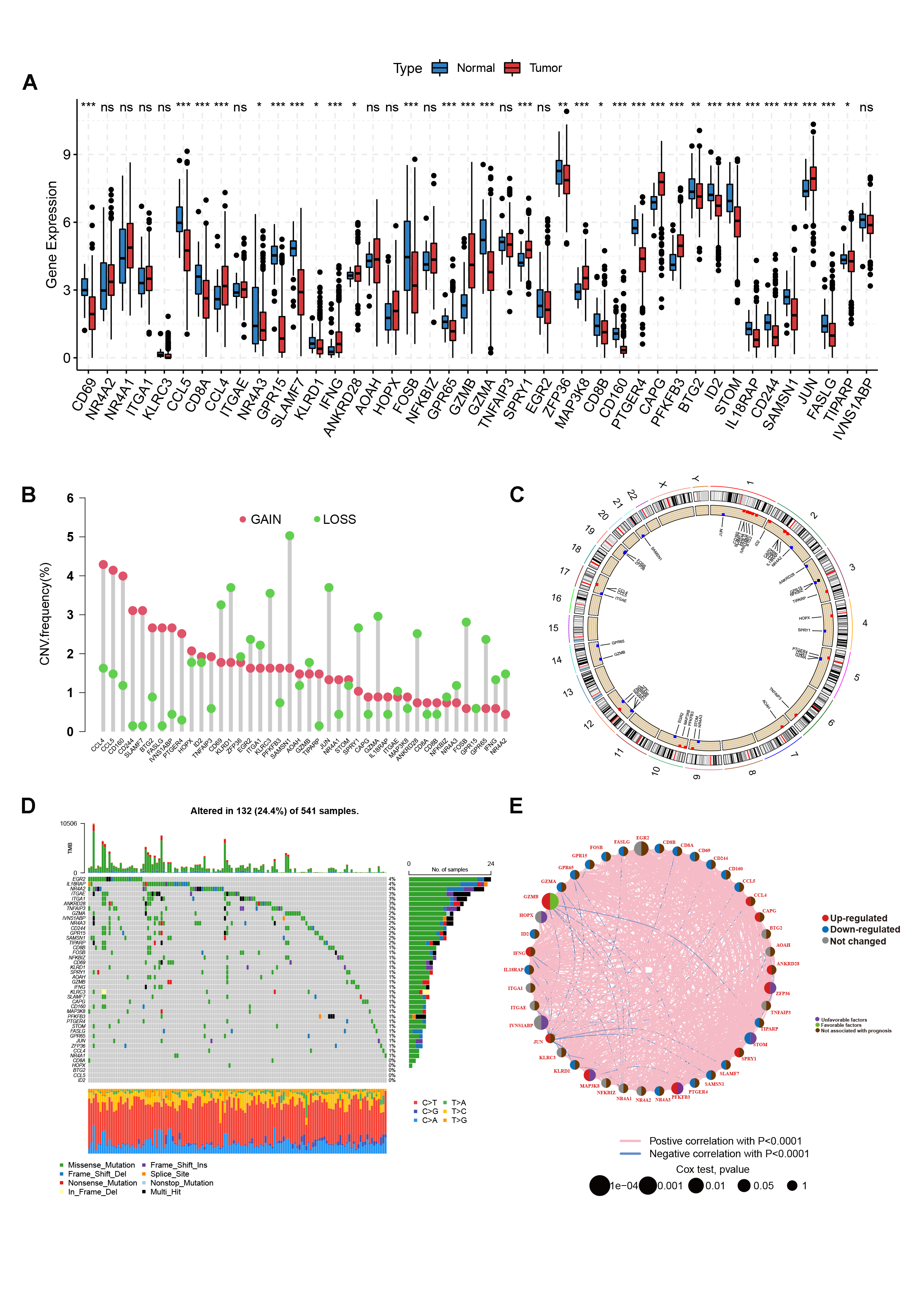

Supplement: Supplementary file 6 [file Image2.TIF]

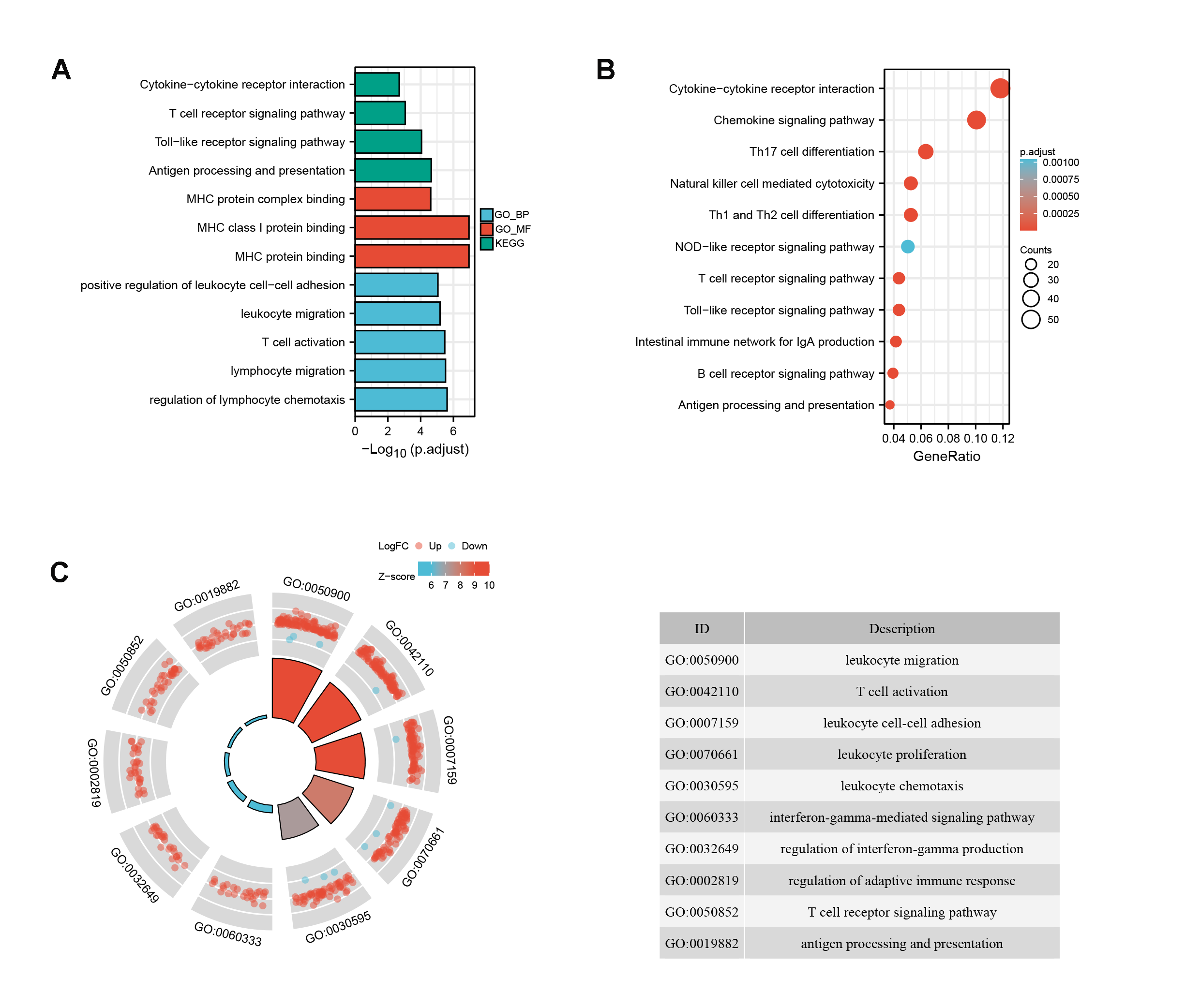

Supplement: Supplementary file 7 [file Image1.TIF]

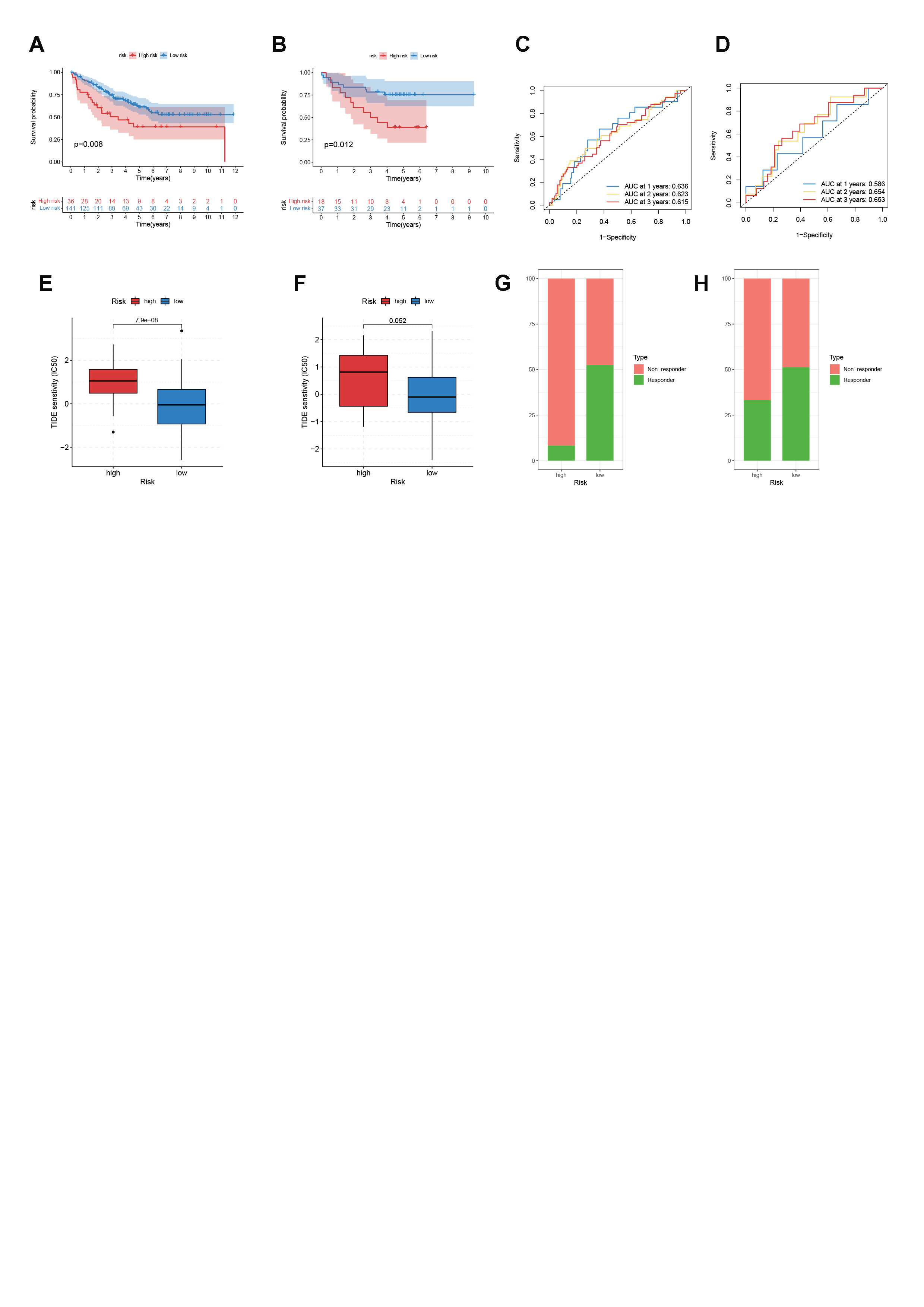

Supplement: Supplementary file 8 [file Image10.TIF]

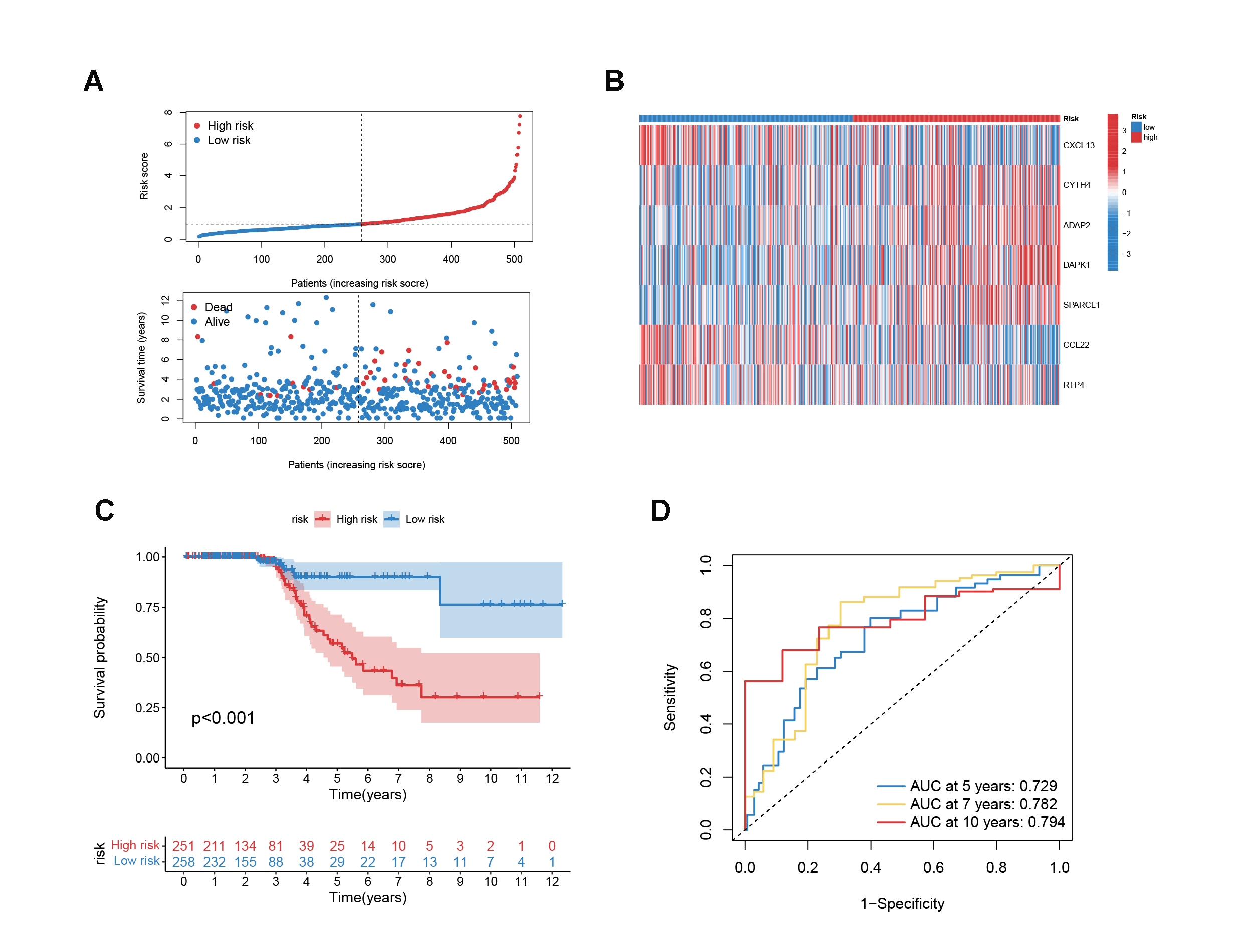

Supplement: Supplementary file 9 [file Image7.TIF]

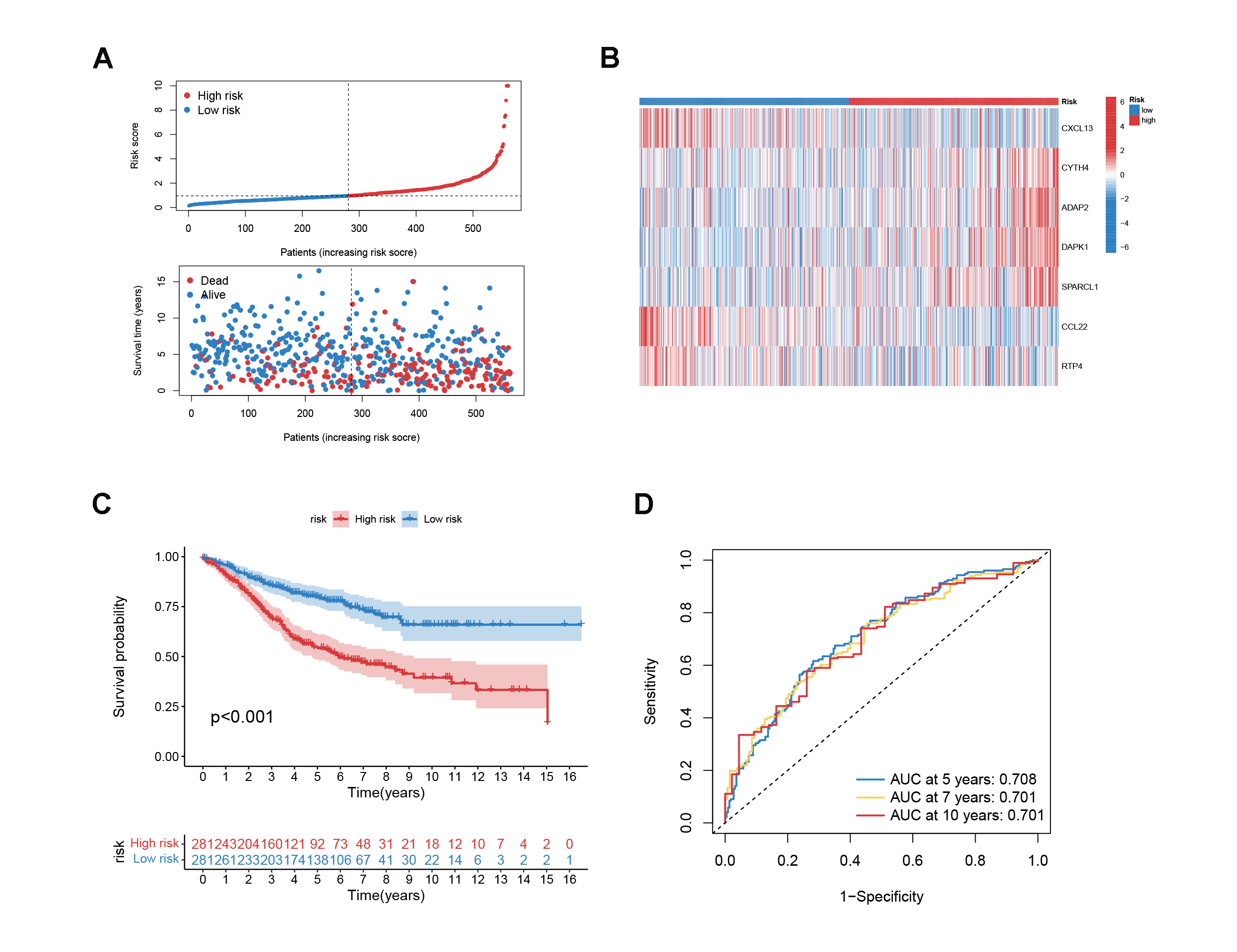

Supplement: Supplementary file 11 [file Image8.TIF]

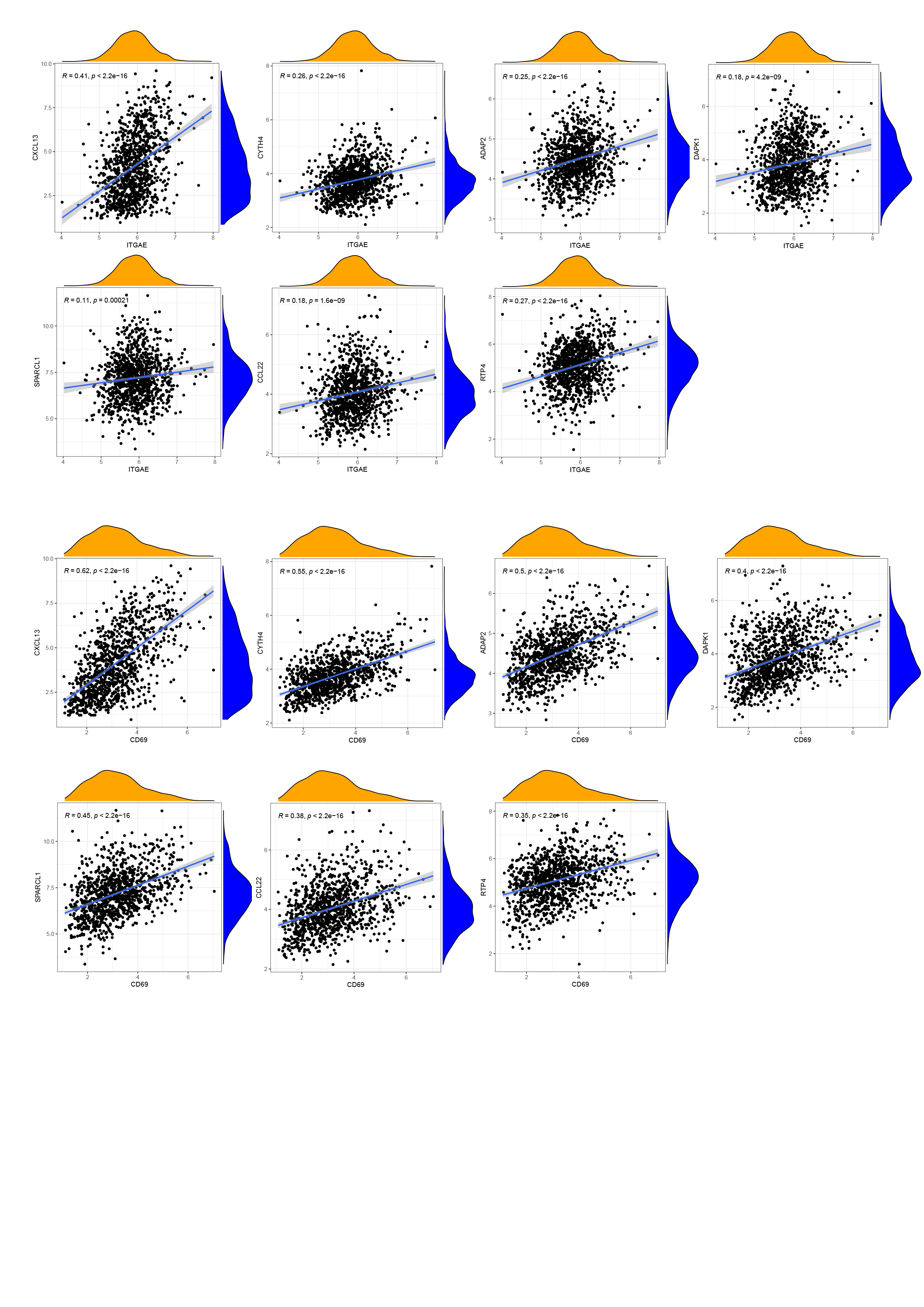

Supplement: Supplementary file 12 [file Image5.TIF]
