# Supplementary material for: Identification of a tissue resident memory CD8 T cell-related risk score signature for colorectal cancer, the association with TME landscapes and therapeutic responses
Source: Front Genet. 2023 Jan 4;13:1088230. doi: 10.3389/fgene.2022.1088230 (PMC9845416; doi:10.3389/fgene.2022.1088230)
Supplement: Supplementary file 13 [file DataSheet1.docx]

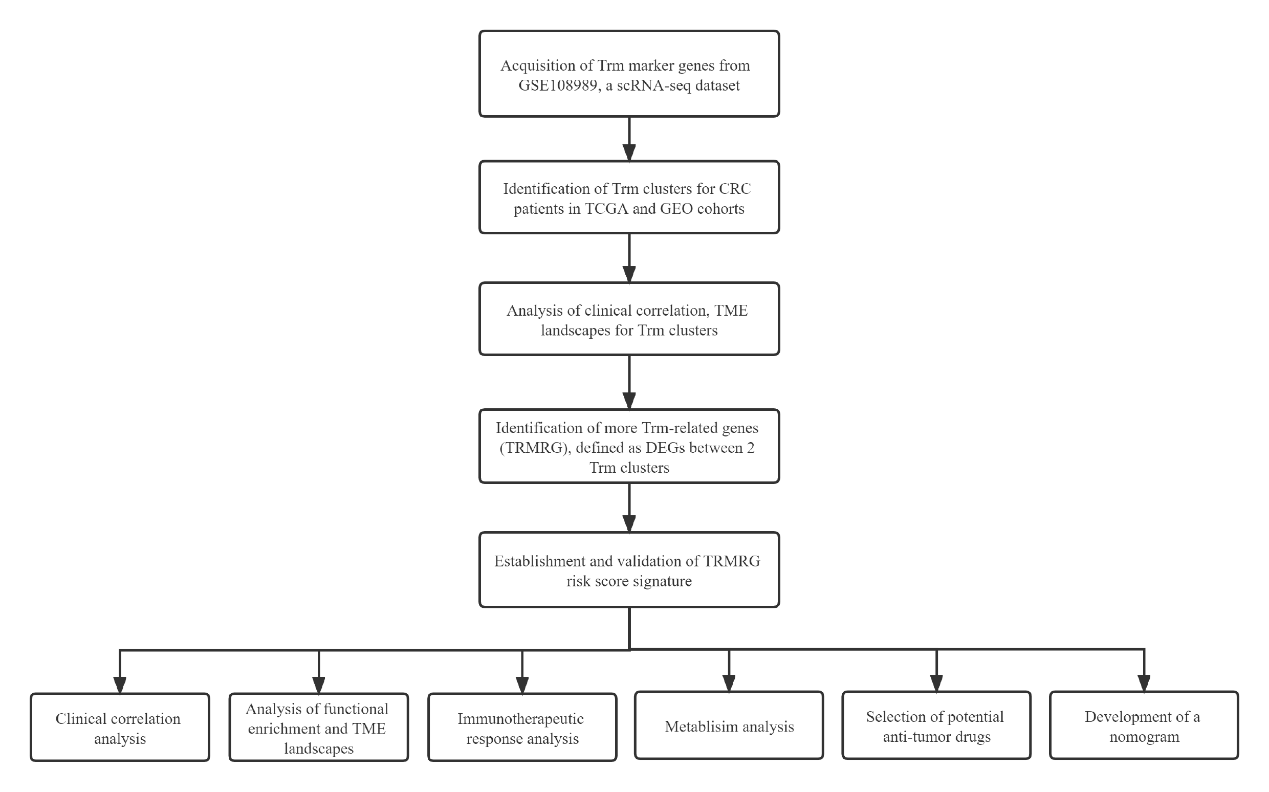


Supplementary Figure S1. Analytical process of the study.


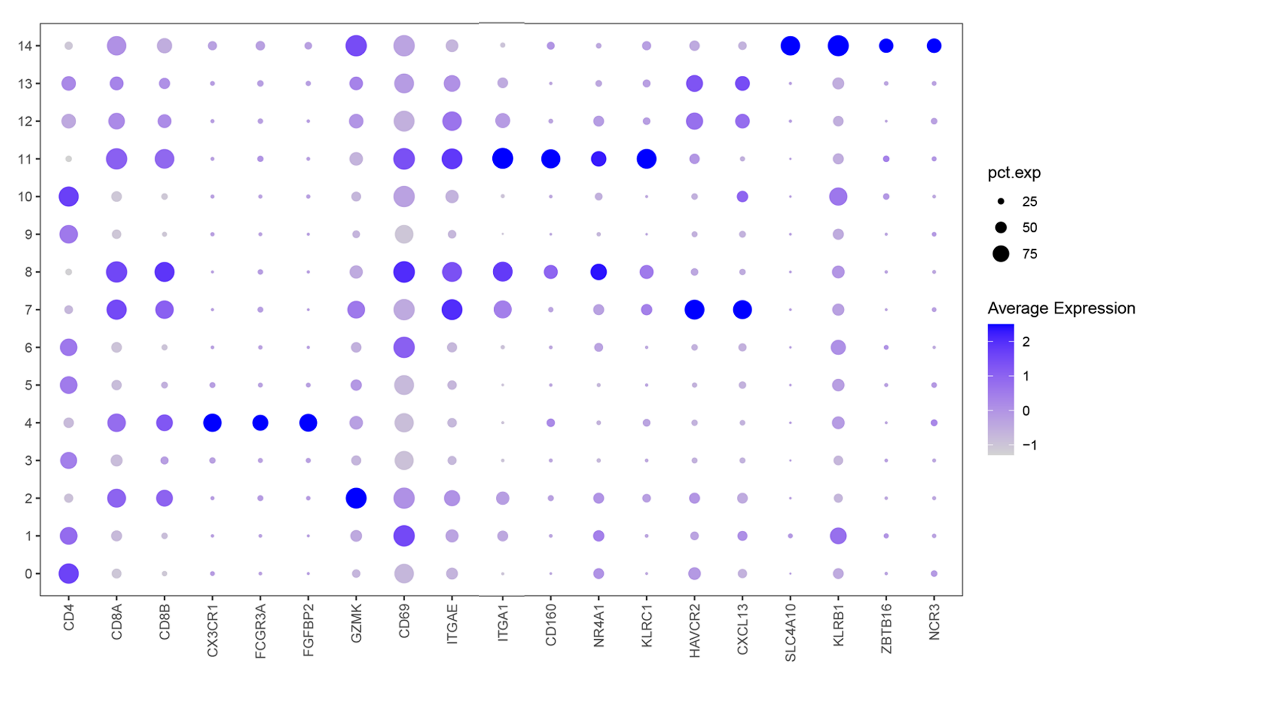


Supplementary Figure S2. The expression of reference genes in different T cell clusters.


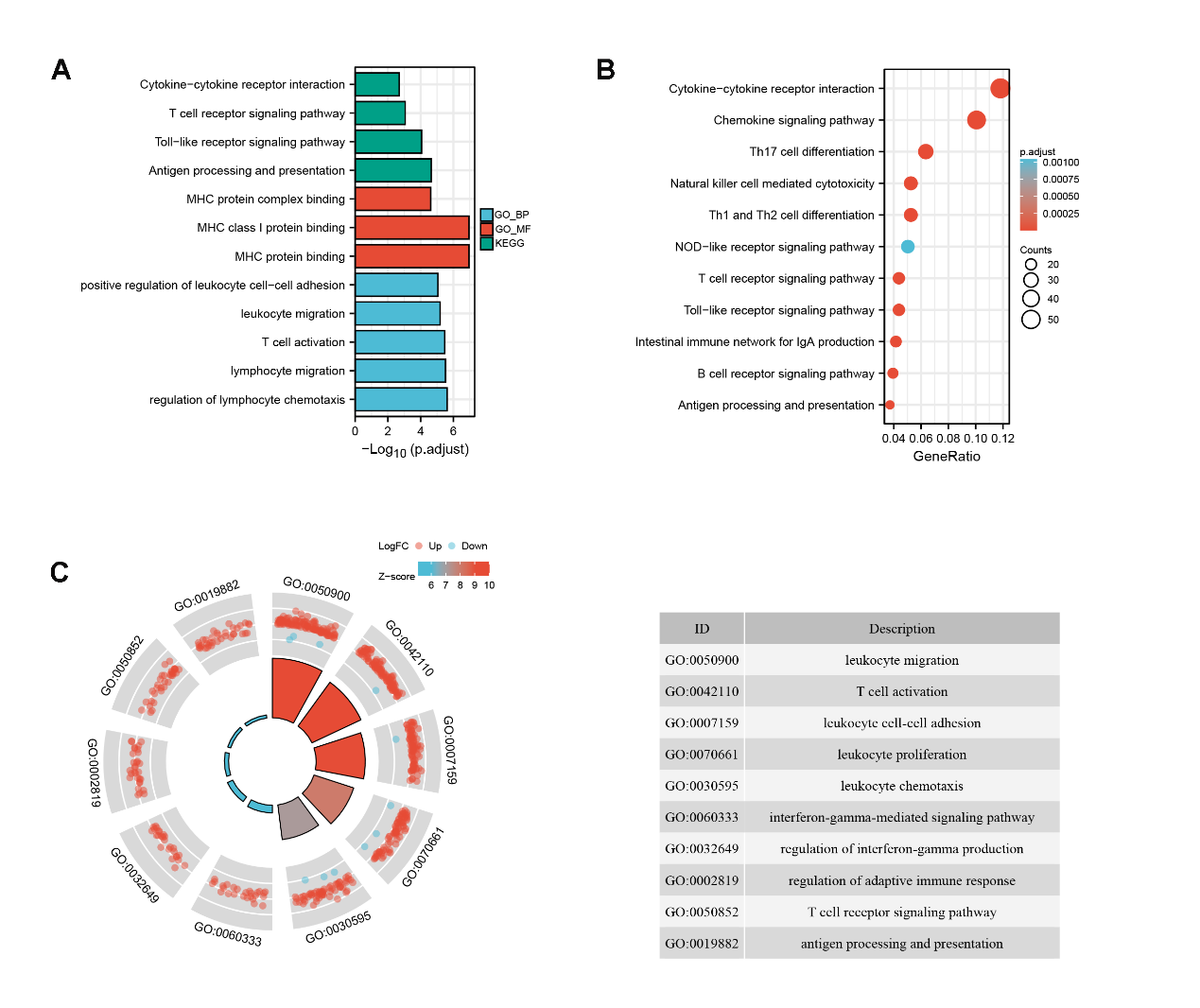


Supplementary Figure S3. Functional enrichment analysis of Trm marker genes and TRMRG. (A) KEGG and GO analysis of Trm marker genes. (B) KEGG and (C) GO analysis of TRMRG. KEGG: Kyoto Encyclopedia of Genes and Genomes; GO: Gene Ontology; MF: molecular function; BP: biological process.


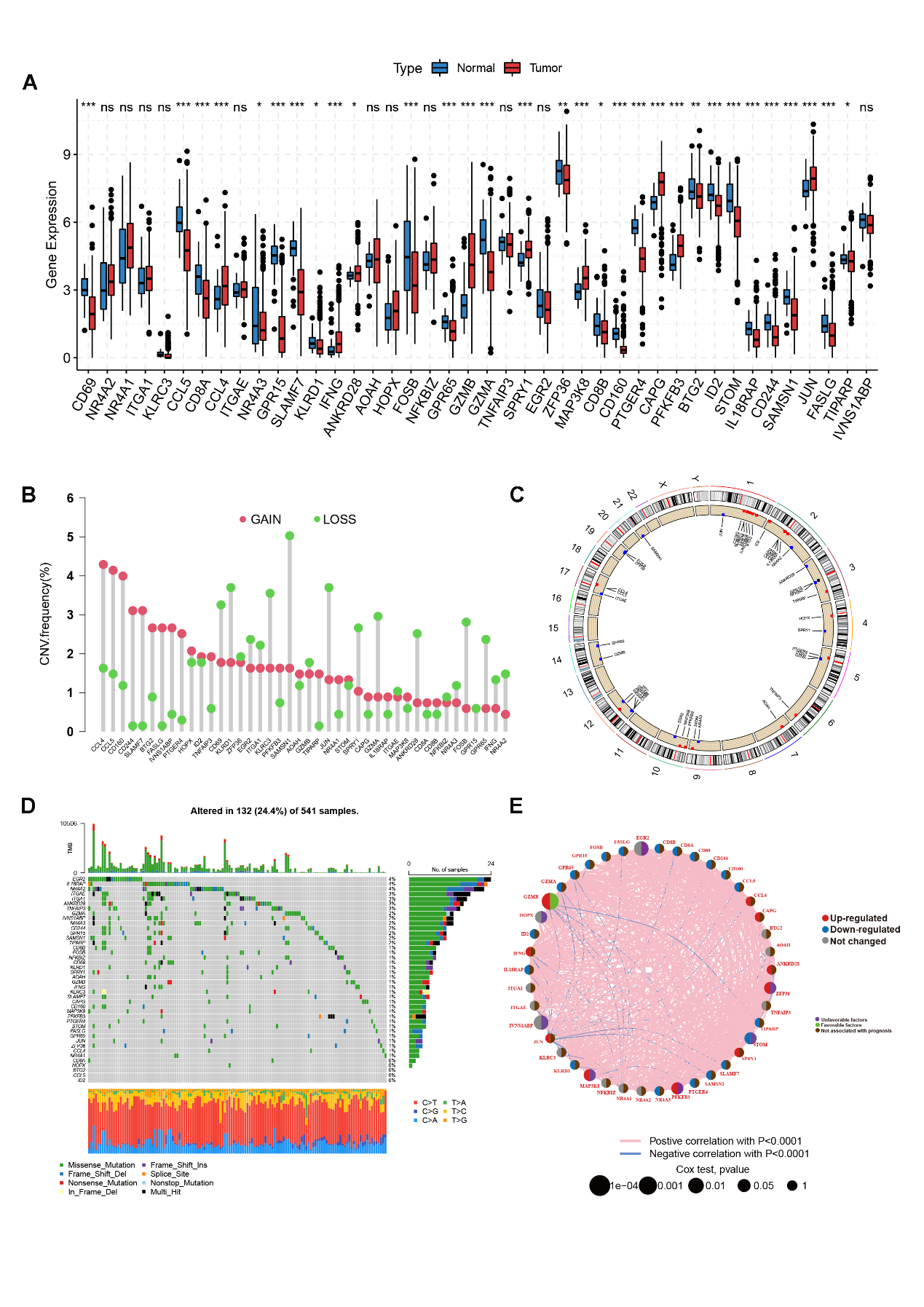


Supplementary Figure S4. Genetic alteration of Trm marker genes. (A) Box plot comparing the expression differences of Trm marker genes between CRC tumor and normal tissues in TCGA cohort. (B) Frequencies of CNV gain and loss for Trm marker genes in TCGA cohort. (C) The chromosomal locations of CNV alterations. (D) Waterfall plot depicting the mutation frequency and mutational subtype of Trm marker genes. (E) Comprehensive network showing the expression correlation and prognostic value of Trm marker genes. Each individual circle represented a single Trm marker gene. The left halves of the circles delineated the expression level of genes, the right halves of the circles delineated the prognostic value of genes. The size of circles represented the p values calculated by univariate Cox analysis. Lines connecting every two circle indicated the interconnection between two genes calculated by Spearman correlation analysis. The thickness of the lines represented the strength of correlation. TMB: tumor mutation burden; CNV: copy number variation. Statistical Significance: *P<0.05; **P < 0.01; ***P < 0.001; ns: not significant.


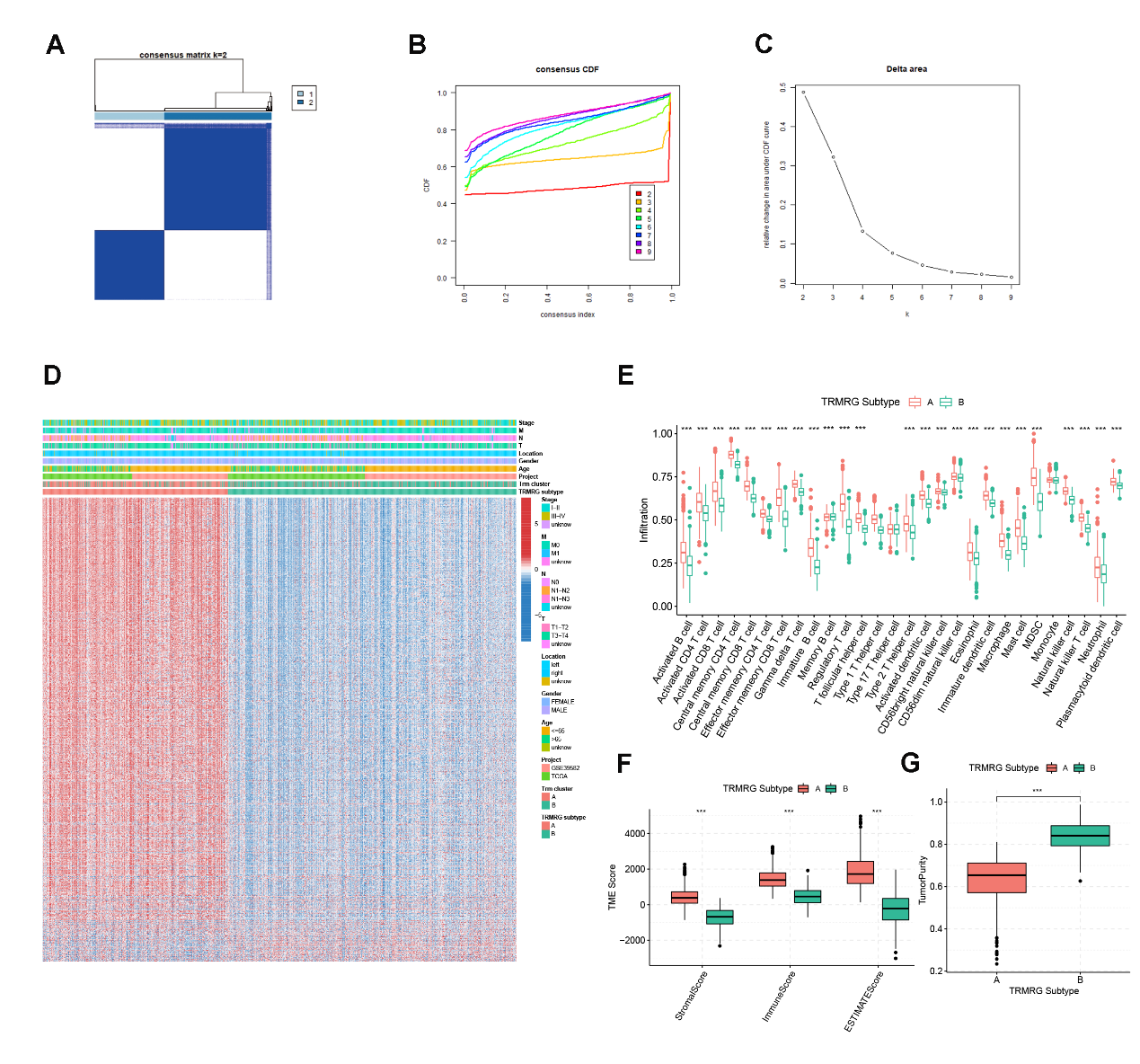


Supplementary Figure S5. Identification of gene subtypes based on TRMRGs. (A) Consensus matrix heatmap showing two gene clusters. (B) CDF curve and (C) k-mean algorithm showing the robustness and stability of the clustering. (D) Heatmap showing the expression pattern of DEGs in two gene subtypes. (E) Box plots comparing the infiltration differences of 28 immune cells between two gene subtypes. (F, G) Box plots comparing the differences in (F) TME scores and (G) tumor purity between two TRMRG subtypes. CDF: Cumulative distribution function; PC: Principal component. Statistical Significance: **P < 0.01; ***P < 0.001.


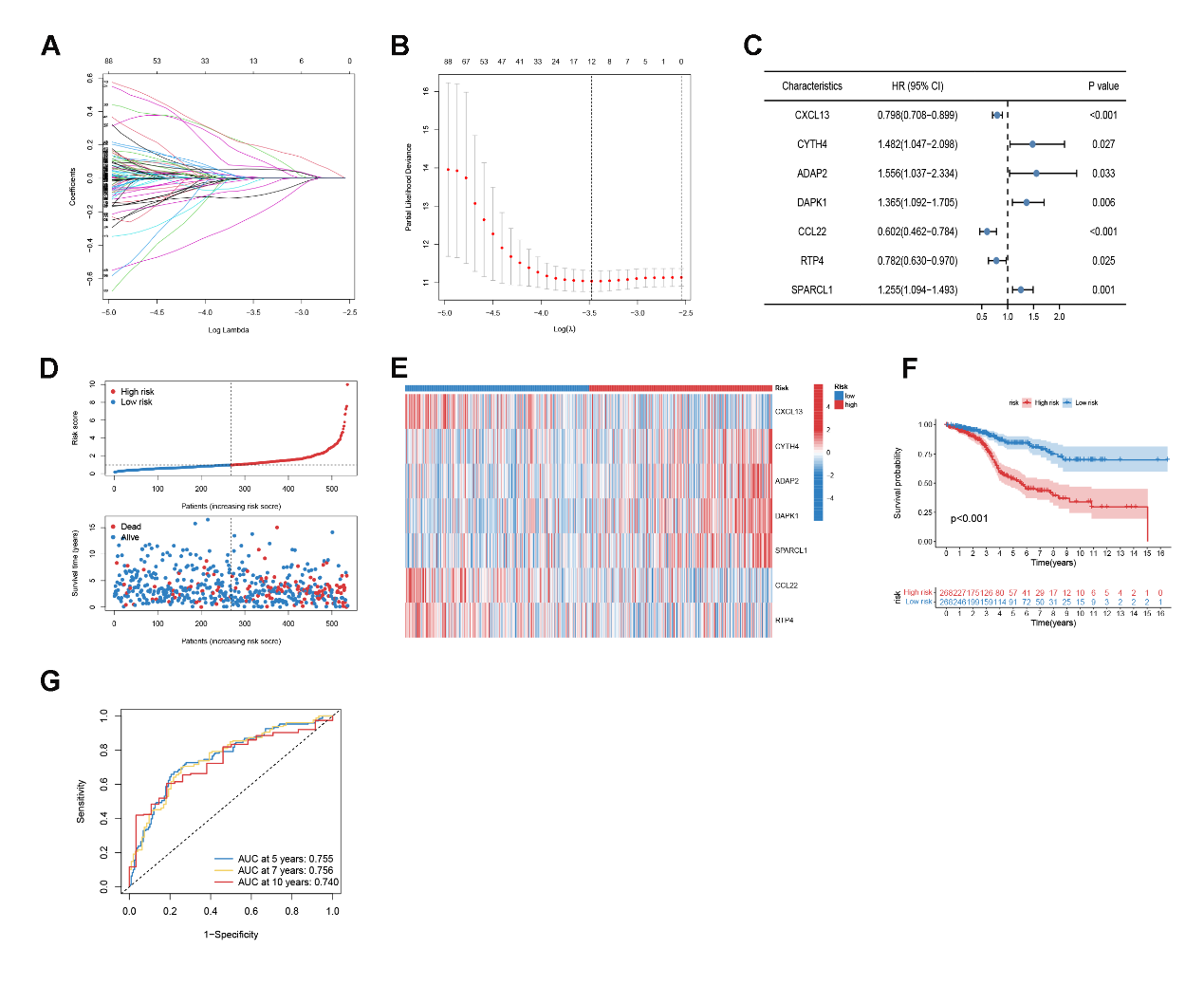


Supplementary Figure S6. Development of TRMRG risk score signature in training cohort. (A, B) The LASSO regression analysis and partial likelihood deviance of prognosis-related DEGs. (C) Multivariate Cox analysis of prognosis-related DEGs. (D) Distribution curve of patients’ risk scores and dot plot of patients’ survival status. (E) Heatmap showing the expression of 7 TRMRGs for high and low risk patients. (F) Kaplan-Meier plot for high and low risk patients. (G) Time-dependent ROC analysis of the risk score for predicting 5, 7 and 10 year OS.


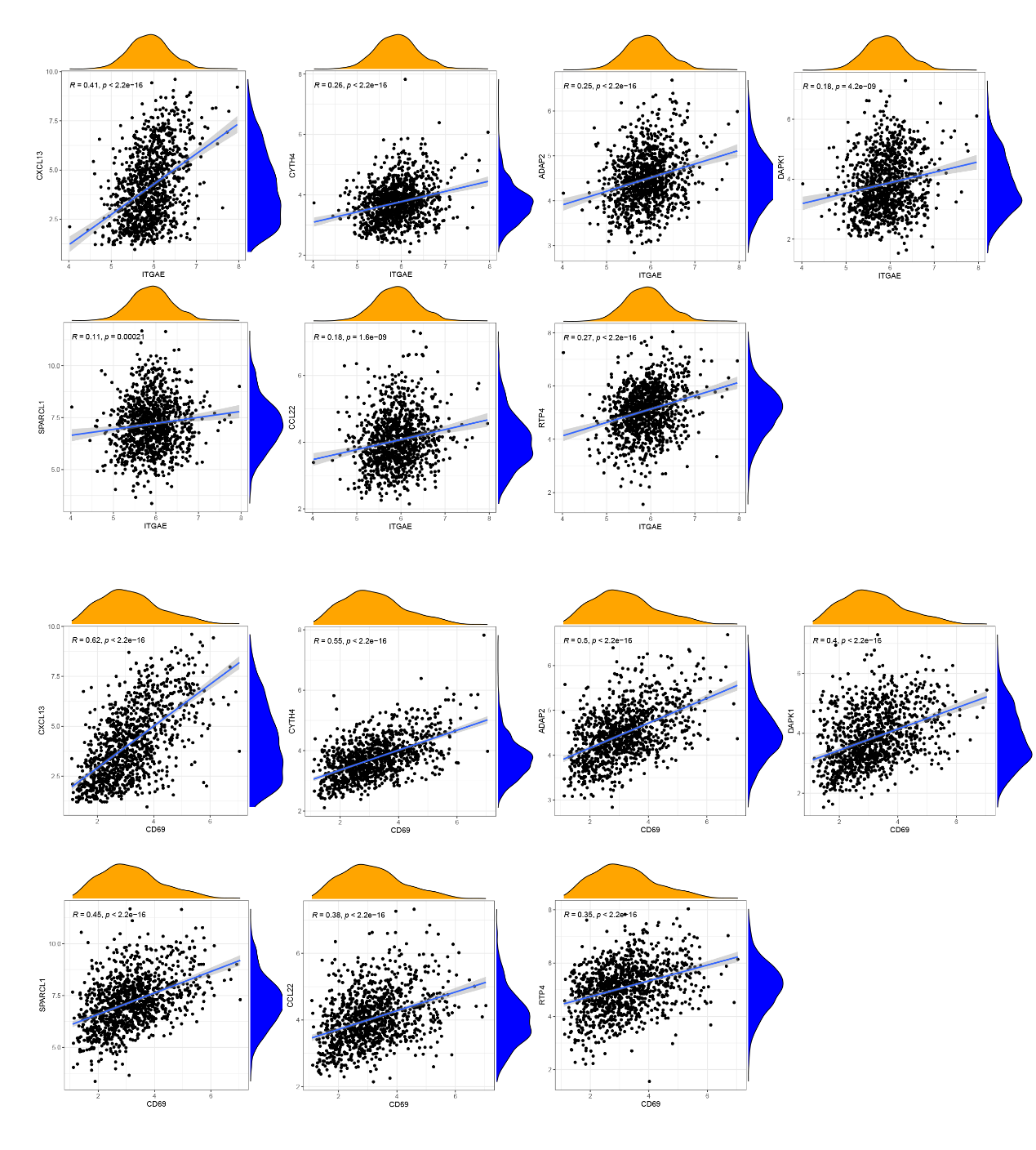


Supplementary Figure S7. Expression correlations between 7 TRMRGs and ITGAE, CD69.


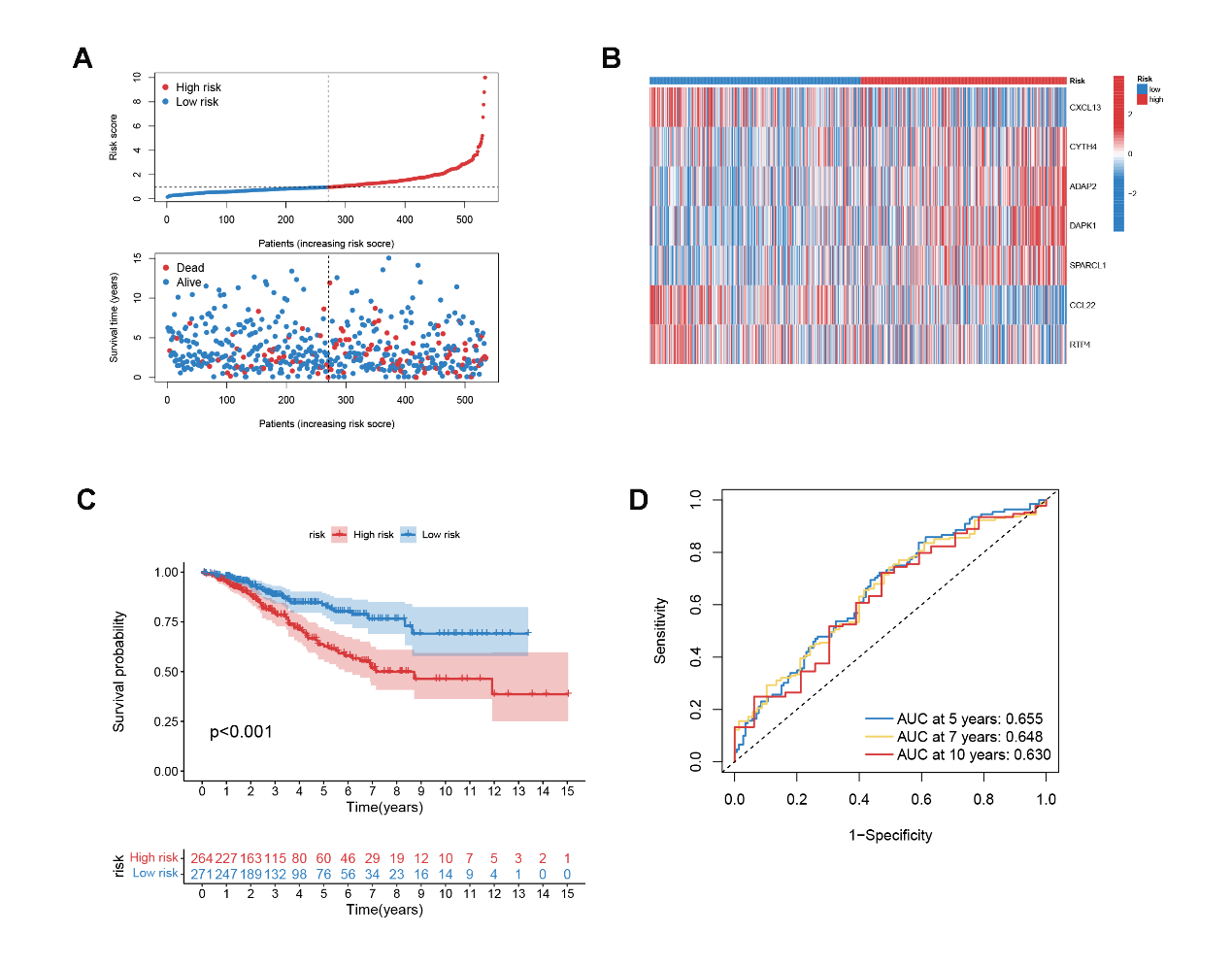


Supplementary Figure S8. Validation of TRMRG risk score signature in test cohort. (A) Distribution curve of patients’ risk scores and dot plot of patients’ survival status. (B) Heatmap showing the expression of 7 TRMRGs for high and low risk patients. (C) Kaplan-Meier plot for high and low risk patients. (D) Time-dependent ROC analysis of the risk score for predicting 5, 7 and 10 year OS.


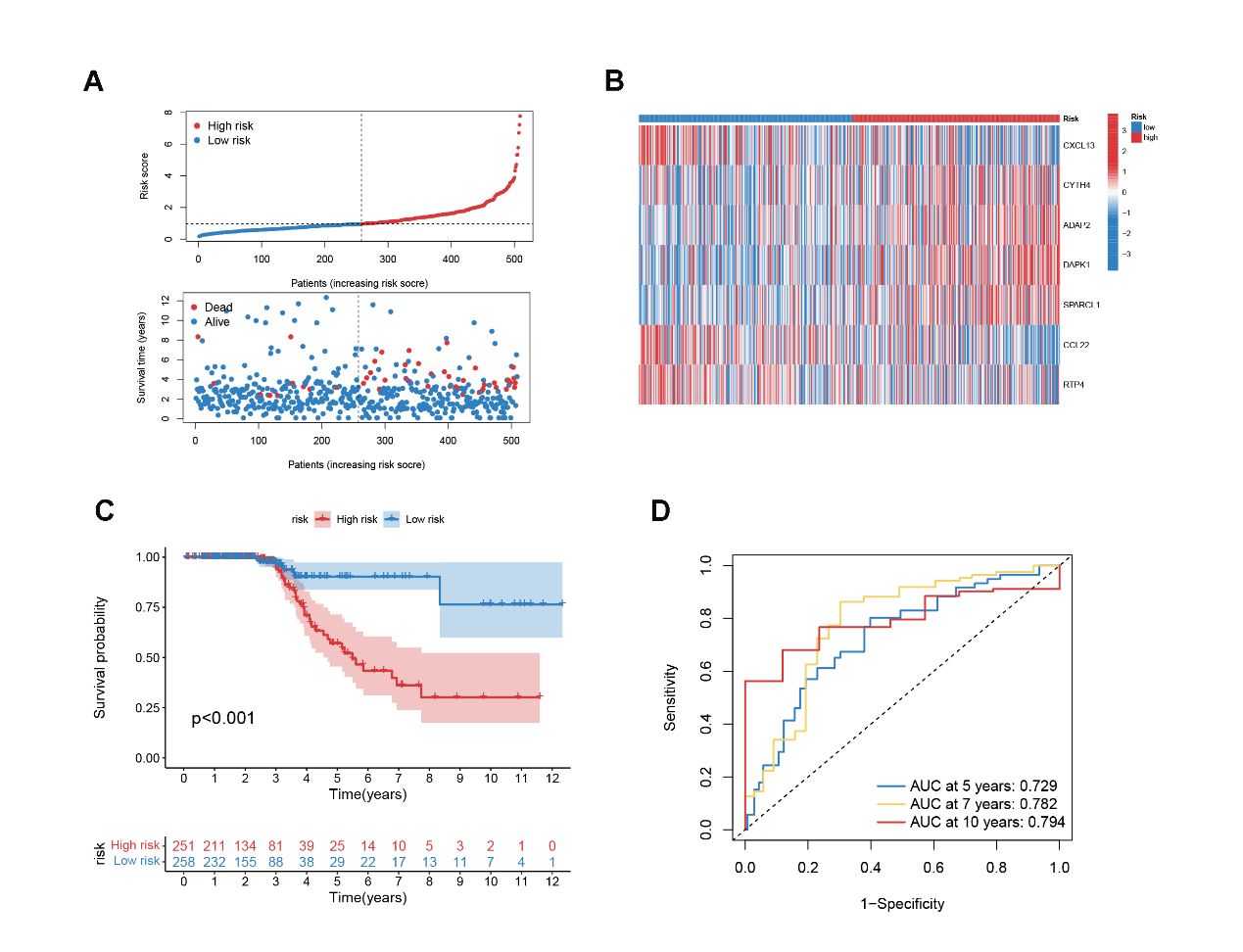


Supplementary Figure S9. Validation of TRMRG risk score signature in TCGA cohort. (A) Distribution curve of patients’ risk scores and dot plot of patients’ survival status. (B) Heatmap showing the expression of 7 TRMRGs for high and low risk patients. (C) Kaplan-Meier plot for high and low risk patients. (D) Time-dependent ROC analysis of the risk score for predicting 5, 7 and 10 year OS.


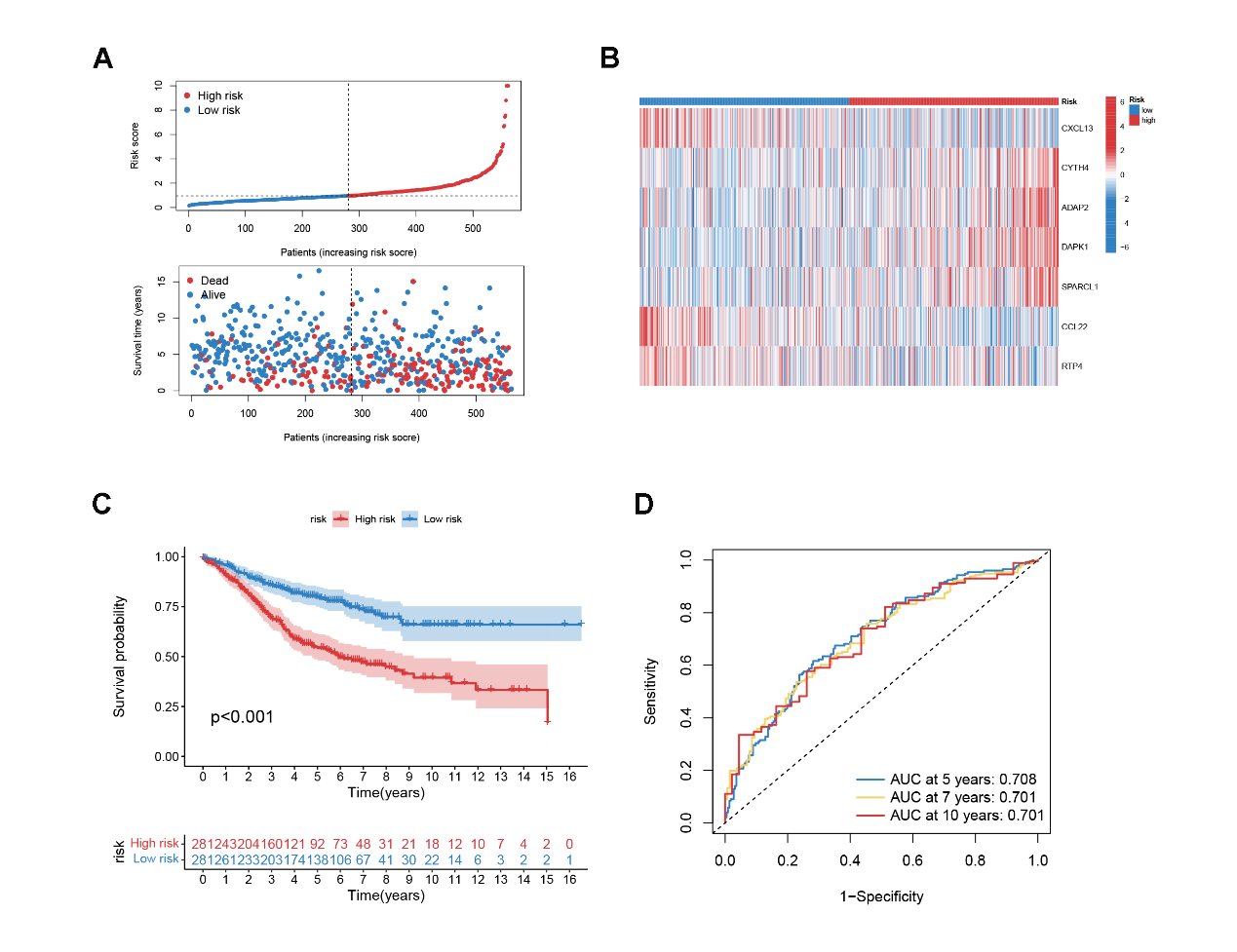


Supplementary Figure S10. Validation of TRMRG risk score signature in GSE39582. (A) Distribution curve of patients’ risk scores and dot plot of patients’ survival status. (B) Heatmap showing the expression of 7 TRMRGs for high and low risk patients. (C) Kaplan-Meier plot for high and low risk patients. (D) Time-dependent ROC analysis of the risk score for predicting 5, 7 and 10 year OS.


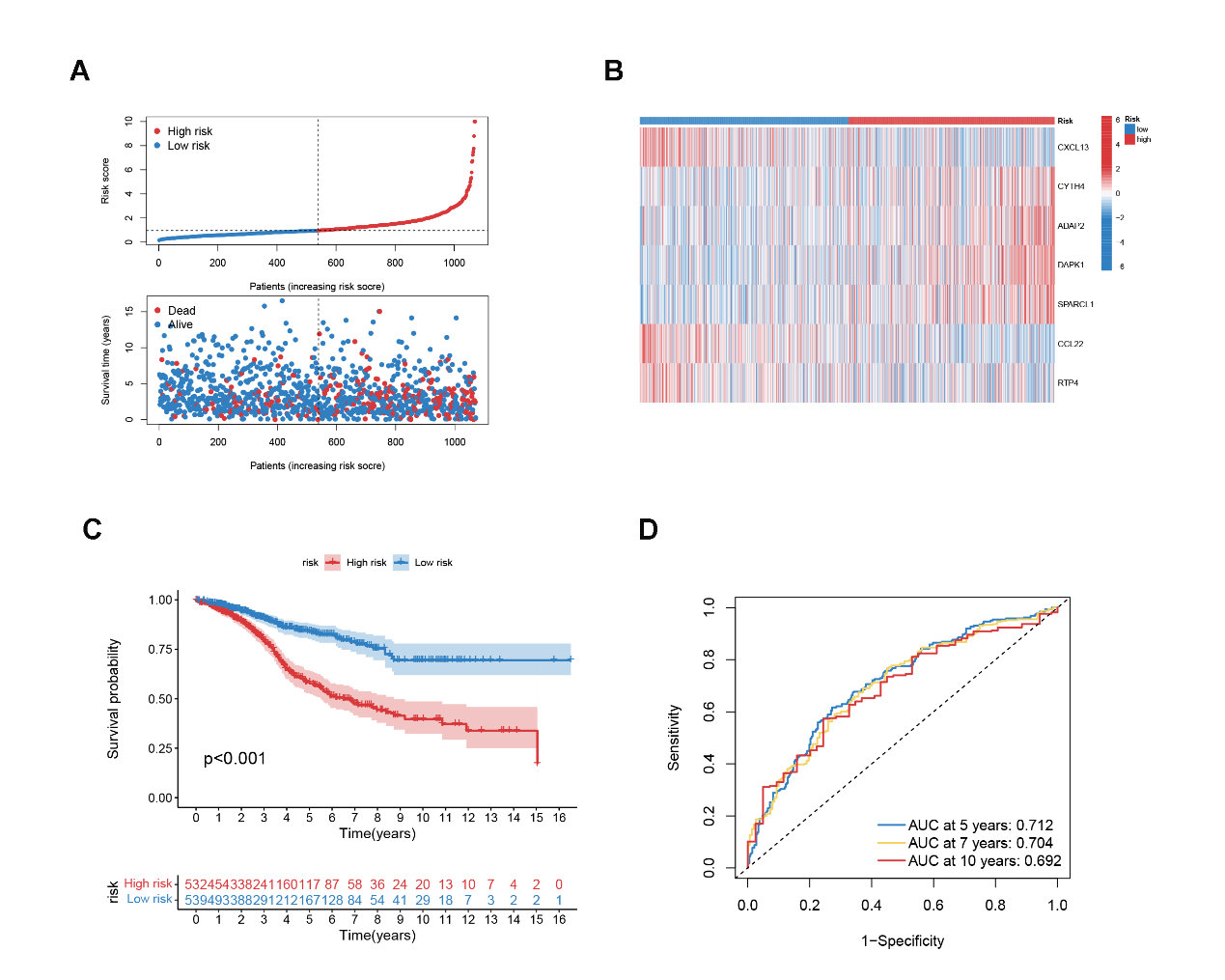


Supplementary Figure S11. Validation of TRMRG risk score signature in meta-cohort. (A) Distribution curve of patients’ risk scores and dot plot of patients’ survival status. (B) Heatmap showing the expression of 7 TRMRGs for high and low risk patients. (C) Kaplan-Meier plot for high and low risk patients. (D) Time-dependent ROC analysis of the risk score for predicting 5, 7 and 10 year OS.


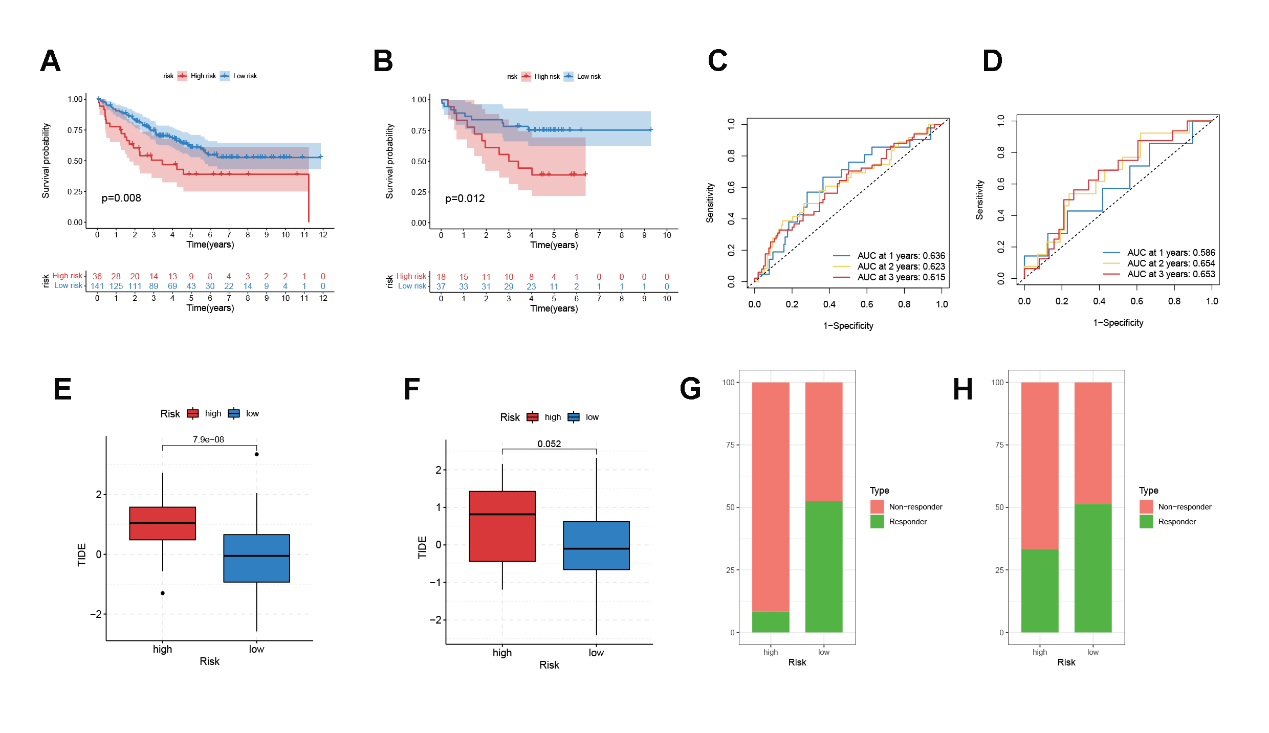


Supplementary Figure S12. External validation of TRMRG risk score signature. (A, B) Kaplan-Meier plots for high and low risk patients in (A) GSE17536 and (B) GSE17537. (C, D) Time-dependent ROC analysis of the risk score for predicting 1, 2 and 3 year OS in (C) GSE17536 and (D) GSE17537. (E, F) TIDE score differences between high and low risk patients in (E) GSE17536 and (F) GSE17537. (G, H) Frequencies of responders and non-responders to ICI therapy in high and low risk patients in (G) GSE17536 and (H) GSE17537. Statistical Significance: **P < 0.01; ***P < 0.001.
